# Calcium Gradient-Doped LiNi_0.5_Mn_1.5_O_4_ Cathode for Long Cycle Life Lithium-Ion Batteries

**DOI:** 10.1021/acsaem.6c00247

**Published:** 2026-04-23

**Authors:** Jie Xiong, Emmanuel Kornyo, Bingyao Zhou, Kevin Mathew, Guoxin Zhang, Wenquan Lu, Zhi Mei, Qingliu Wu

**Affiliations:** a Department of Chemical and Paper Engineering, 4175 Western Michigan University, 4601 Campus Drive, Kalamazoo, Michigan 49008-5462, United States; b Chemical Science and Engineering Division, Argonne National Laboratory, 9700 South Cass Ave., Lemont, Illinois 60439-4837, United States; c Lumigen Instrument Center, 2954 Wayne State University, 5101 Cass Ave, Detroit, Michigan 48202, United States

**Keywords:** high-voltage spinel, gradient doping, calcium, lithium-ion battery, cation disorder

## Abstract

High-voltage spinel LiNi_0.5_Mn_1.5_O_4_ (LNMO) has attracted considerable attention as a cathode material for next-generation lithium-ion batteries due to its high operating voltage and intrinsically fast lithium-ion diffusion kinetics. However, the practical implementation of LNMO remains limited by its rapid capacity decay, primarily associated with bulk structural instability and parasitic interfacial reactions. To address these issues, we innovatively introduced calcium (Ca) as a dopant to enhance both the oxygen framework and surface stabilities of the LNMO crystal through gradient doping. Observations from the electronic microscopies, X-ray diffraction, and the elemental analysis confirmed that Ca is preferentially enriched at the particle surface, and a disordered crystal phase is preserved in the bulk in the gradient-doped LNMO cathodes. As cathodes in LIBs, the Ca gradient-doped (Ca gr) LNMO materials delivered formation capacities of ∼126–130 mAh/g and exhibited Coulombic efficiencies of 88–95%, which are consistently higher than those of the uniform-doped samples at the same doping level and undoped sample. Especially, the Ca gr 0.05 LNMO cathode demonstrated significantly improved rate capability with ∼113 mAh/g preserved at 10 C, while ∼92 mAh/g and ∼110 mAh/g for undoped and Ca uniform 0.05 LNMO, respectively, and excellent cycling stability, retaining ∼124.1 mAh/g (∼96.3% capacity retention) after 500 cycles. The analysis of cyclic voltammetry, differential capacity, and electrochemical impedance revealed that the excellent electrochemical performance is attributed to the structural and morphological advantages of gradient-doped LNMO cathodes with a disordered bulk structure for fast Li^+^ diffusion and a Ca-enriched surface for minimizing the Mn dissolution.

## Graphical abstract

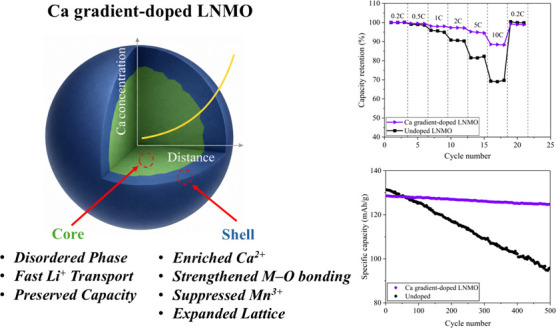

## 1 Introduction

The high-voltage spinel material with the formula of LiNi_0.5_Mn_1.5_O_4_ (LNMO) is widely regarded as one of the most promising cathode materials for next-generation lithium-ion batteries (LIBs), primarily due to its cobalt-free composition, high operating voltage (∼4.7 V vs Li/Li^+^), and rapid lithium-ion transport enabled by its three-dimensional spinel structure.^1,2^ However, the practical application of LNMO is still hindered by its severe capacity fading at high temperatures, driven by the combined bulk structural instabilities (e.g., oxygen release and Jahn–Teller distortion) and interfacial side reactions (e.g., Mn dissolution and cathode-electrolyte interface (CEI) film thickening) with liquid electrolytes.^3–8^ Consequently, strategies that can simultaneously enhance both structural and interfacial stability are essential to achieve durable long-term electrochemical performance in LNMO cathode materials.

Numerous strategies have been investigated to mitigate the surface and structural degradation of LNMO cathodes during cycling. Surface modification, particularly through the deposition of thin protective layers, has been widely adopted to limit direct electrolyte exposure at high operating potentials, thereby suppressing interfacial parasitic reactions and stabilizing the electrode–electrolyte interface.^5–7^ For example, Kim et al. demonstrated that depositing an ultrathin Al_2_O_3_ layer (<1 nm) via atomic layer deposition led to significantly improved capacity retention of LNMO electrodes, reaching 98% after 150 cycles compared to approximately 94% for untreated samples.^8^ The improved cyclability was ascribed to the suppressed side reactions at high voltage and reduced transition-metal dissolution. Similar interfacial stabilization effects have also been reported for surface coatings with alternative materials.^9,10^ Although surface coatings can mitigate parasitic reactions and limit excessive growth of the cathode–electrolyte interphase (CEI), overly thick or poorly conductive layers may reduce active material utilization and hinder Li^+^ transport. More importantly, surface modification alone does not address the intrinsic structural instability of LNMO, which continues to drive capacity decay during prolonged cycling.^6^ Accordingly, these limitations have prompted increased interest in elemental doping strategies that stabilize the LNMO lattice without compromising its high energy density.

Various heteroatomic elements, such as Cr, Fe, F, and Cl, have been incorporated into the spinel structure to mitigate structural degradation during repeated electrochemical cycling of LNMO cathodes. For example, Cr-substituted LNMO (Cr0.1 LNMO) demonstrated superior cyclability, retaining more than 94% of its capacity after 500 cycles, compared with ∼82% retention for pristine LNMO.^11^ The improved stability of the Cr-doped LNMO cathode was attributed to the suppressed Mn^3+^ formation, mitigated Jahn–Teller distortion, and stabilized spinel framework by strengthened Cr–O bonds. Similar improvements in electrochemical performance have also been reported for LNMO cathodes doped with elements such as Ti, Fe, Nb, and Mg.^10–12^ Our previous work^13^ also showed that the dopants of Sr, Cr, and Ca with ionic radius larger than those of Ni and Mn can boost not only the cyclability, but also the rate capability of LNMO cathodes through the suppressed Mn^3+^ content and improved structural stability. We found that the bonding strength of dopants to oxygen plays an important role in determining the structural stability of LNMO crystals. In addition, the durability, or the structural stability, of LNMO cathodes increases with the enhancement of the Ca content. Despite these benefits, the elemental doping strategy typically involves partial substitution of redox-active Ni/Mn ions with electrochemically inactive dopants, and excessive incorporation can significantly reduce the reversible capacity of LNMO cathodes.^7^

Gradient doping has gained increasing attention as an advanced compositional design strategy that integrates the advantages of surface stabilization with the preservation of bulk redox activity.^14^ In this strategy, dopant species are preferentially enriched at the particle surface and gradually decrease toward the interior, enhancing interfacial robustness while preserving a largely intact, redox-active core. This method, therefore, can effectively stabilize both the surface and bulk structure of cathode materials while minimizing the capacity penalty associated with uniform doping. The advantages of the concentration gradient design have been extensively validated in Ni-rich layered cathodes. For instance, Liu et al. designed Ni-rich cathodes with a radially graded Ni/Co/Mn distribution that exhibited largely improved cycling durability, maintaining >90% capacity retention after 200 cycles, compared to only ∼70% for the uniform counterpart.^15^ Zeng et al. further demonstrated that gradient-designed Ni-based cathodes demonstrated a high rate capability (∼180 mAh/g at 5C), while retaining 85% of their initial capacity after 500 cycles, significantly outperforming homogeneous composition.^16^ The superior performance of such gradient structures can be attributed to their tailored elemental distribution. The Ni-rich core preserves high energy density, while the Mn/Co-enriched shell suppresses parasitic surface side reactions, mitigates structural degradation, and alleviates strain accumulation during cycling. It is believed that the surface-enriched compositions mitigate electrolyte attack while the core preserves high-capacity Ni redox activity, leading to improved cycling stability.^15–17^

Despite its promises, few studies have been reported to investigate the potential of the gradient doping approach in improving the performance of LNMO cathodes. Liu et al. reported that Mg gradient-doped LNMO, which was prepared via the coprecipitation process, exhibited the superior rate capability (∼117 mAh/g at 4 C) and cycling stability (92% capacity retained after 80 cycles), compared to uniformly doped counterparts (81 mAh/g at 4 C and 80% capacity retained after 80 cycles).^18^ Luo et al. reported a fluorine gradient doping strategy for LNMO, where F was enriched at the surface without altering the bulk spinel structure.^9^ The surface M–F bonding and LiF-rich CEI effectively suppressed electrolyte oxidation, oxygen release, and Mn dissolution, leading to improved high-voltage stability and slower impedance growth.^9^ Motivated by prior studies on gradient-doped cathode materials, we propose that a Ca concentration gradient design can simultaneously enhance surface and bulk stability in LNMO while minimizing the loss of specific capacity. Calcium was specifically selected as the dopant over other common elements (e.g., Mg and Al) due to its exceptionally strong bonding affinity to oxygen and its large ionic radius (1.00 Å).^19,20^ While uniformly doping such large ions induces severe bulk lattice strain and cation ordering,^21^ this significant size mismatch makes Ca uniquely suited for a gradient approach. The structural flexibility of the particle surface could accommodate the large Ca^2+^ ions, effectively confining the dopant to the outer layers. Therefore, it is rational to expect that, if appropriately incorporated, the Ca gradient-doped LNMO will utilize strong Ca–O bonds to provide a chemically resilient surface against the electrolyte attack, while preserving a disordered spinel bulk for high specific capacity.

In this work, the Ca element was introduced into LNMO as a dopant by using a controlled gradient-doping strategy designed to enrich Ca at the surface of LNMO particles while minimizing bulk incorporation. The structural properties of the Ca-gradient-doped LNMO were comprehensively investigated using X-ray diffraction (XRD), electron microscopies (SEM and TEM), and electron probe analysis. The effect of Ca gradient doping on the electrochemical performance of LNMO cathodes was systematically evaluated in the LIBs. The underlying mechanisms responsible for the improved electrochemical performance of Ca-gradient-doped LNMO cathodes were also investigated.

## 2 Experimental Section

### 2.1 Chemicals

Manganese nitrate hydrate (Mn­(NO_3_)_2_•xH_2_O, ≥98%, Fisher Chemical), nickel nitrate hexahydrate (Ni­(NO_3_)_2_•6H_2_O, ≥98%, Fisher Chemical), sodium hydroxide (NaOH, ≥98%, Fisher Chemical), calcium nitrate tetrahydrate (Ca­(NO_3_)_2_, 99+%, ACS reagent), 1-methyl-2-pyrrolidone (NMP, 99%, Acros Organics), carbon black (C45, Timcal), polyvinylidene difluoride (PVDF 5130, molecular weight of 1300 kDa, Solvay), lithium monohydrate (LiOH•H_2_O, 99%, Thermo Fisher Scientific), aqueous ammonia solution (NH_3_•H_2_O, 28–30 wt %), coin cell cases (CR2032, Canrd), and microporous membrane (39% porosity, Celgard) were used as received without further treatment.

### 2.2 Material Synthesis

Ca gradient-doped LNMO (Ca gr *x*, *x* = 0.01–0.1) was synthesized through a two-step route involving coprecipitation of Ni/Mn precursors with gradually increased Ca addition, followed by high-temperature calcination with lithium salt. In a typical synthesis of Ca gradient-doped LNMO precursors, three solutions were prepared: (1) solution A containing manganese nitrate (Mn­(NO_3_)_2_) and nickel nitrate (Ni­(NO_3_)_2_) dissolved in deionized water at a stoichiometric ratio, and (2) solution B consisting of a mixed base solution of 4 M NaOH and 1 M NH_4_OH, and (3) solution C containing Ca­(NO_3_)_2_. The concentration of Ca­(NO_3_)_2_ in solution C was varied to achieve different Ca gradient doping levels. During coprecipitation, solution C was continuously introduced into solution A. The resulting mixture solution of A and C was fed into a 32 mL 1 M NH_4_OH mother solution maintained at 60 °C, while solution B was introduced simultaneously to maintain the pH at ∼11. This configuration allowed Ca^2+^ to be gradually introduced during the continuous growth of the precursor particles, generating a radial Ca concentration gradient from the particle core to the surface. The formation of the Ca concentration gradient is closely related to the time-dependent growth of the precursor particles during coprecipitation. Since the precursor particles continuously nucleate and grow from the interior toward the exterior, the early-formed particle cores are exposed to a relatively lower Ca^2+^ concentration, whereas the later-formed outer regions encounter a progressively higher Ca^2+^ concentration as solution C is continuously added. As a result, Ca is preferentially incorporated into the outer region of the precursor particles, leading to surface-enriched gradient doping after calcination. The feeding rate of solution C and the stirring intensity are expected to influence the extent of the gradient. The feeding rate of the Ca precursor influences the Ca^2+^ concentration during coprecipitation, and a controlled introduction of Ca^2+^ allows particles formed at different growth stages to incorporate different amounts of Ca, promoting the formation of a radial concentration gradient.^22,23^ Stirring rate also influences gradient formation. Excessive mixing can homogenize the solution and reduce local concentration differences, therefore weakening the gradient distribution.^23,24^ In this study, the feeding rate and stirring rate were optimized during preliminary experiments and kept constant for all gradient-doped samples to ensure reproducible formation of the Ca-enriched surface structure. After the reaction, the suspension was aged for 2 h, filtered, washed with DI water to neutral pH, and dried overnight at 70 °C in air. The obtained precursors were mixed with LiOH at a molar ratio of Li:(Ni + Mn + Ca) = 1.05:1, followed by grinding in an agate mortar for ∼30 min to ensure homogeneous mixing. The mixture was then put into a crucible and placed inside a tube furnace for a two-step thermal treatment: an initial calcination at 900 °C for 15 h and a subsequent annealing step at 700 °C for 8 h, both in air flow. The heating ramp rate was maintained at 5 °C/min for the entire process.

For comparison, Ca uniform-doped LNMO (Ca uniform-*x*, *x* = 0.01–0.1) samples with the same nominal Ca contents as the gradient-doped LNMO were synthesized using a conventional coprecipitation route involving two solutions. Solution A contained Mn­(NO_3_)_2_, Ni­(NO_3_)_2_, and Ca­(NO_3_)_2_ dissolved together in deionized water, while solution B, a mixed base of 4 M NaOH and 1 M NH_4_OH, was identical to that used for the Ca gradient-doped synthesis. Solution A was coprecipitated with solution B under identical conditions (60 °C, pH ≈ 11), leading to uniform Ca incorporation throughout the precursor particles. All subsequent processing steps, including aging, washing, drying, lithium addition, and calcination, were kept the same as those used for the Ca gradient-doped LNMO to enable a direct comparison.

### 2.3 Material Characterizations

#### Crystallinity

The crystal structure of the LNMO was characterized by X-ray diffractometer (XRD, Rigaku SmartLab SE) with Cu Kα radiation (λ = 1.5406 Å).

#### Morphology and Microstructure

The surface morphology of the synthesized materials was investigated by scanning electron microscopy (SEM, JEOL IT200) operated at accelerating voltages ranging from 1 to 30 kV. High-resolution transmission electron microscopy (HRTEM) and selected-area electron diffraction (SAED) analyses were performed using a Thermo Fisher Talos F200X G2 S/TEM instrument.

#### Chemical Composition and Elemental Distribution

The distribution of Ca within LNMO particles was characterized by scanning electron microscopy coupled to energy-dispersive X-ray spectroscopy (SEM-EDS) and electron probe microanalysis (EPMA). For cross-sectional analysis, LNMO powders were embedded in an epoxy resin and cured at room temperature overnight. The hardened specimens were subsequently sectioned, mechanically ground, and polished to expose smooth particle cross sections for compositional mapping.

### 2.4 Electrode Fabrication and Cell Assembly

#### Electrode Fabrication

Undoped, Ca uniform-doped, or Ca gradient-doped LNMO powders were first mixed with carbon black (C45) as the conductive additive. The resulting powder mixture was then dispersed in a poly­(vinylidene fluoride) (PVDF) binder solution (8 wt % PVDF in *N*-methyl-2-pyrrolidone, NMP) to form a slurry. Additional NMP was introduced to adjust the slurry viscosity, yielding an overall solid content of ∼40 wt %. The slurry was uniformly coated onto aluminum foil current collectors using a doctor blade and subsequently dried at 40 °C for 4 h to allow controlled solvent evaporation. The mass loading of the active material was controlled at ∼2 mg/cm^2^. The electrodes were further dried under vacuum at 120 °C overnight to remove residual NMP. All dried electrodes have the composition of 80 wt % active material, 10 wt % conductive additive, and 10 wt % binder.

#### Cell Assembly

The dried electrodes were punched into circular disks (9/16 in. in diameter) and assembled into CR2032 coin cells by using lithium metal foil as the counter electrode. The electrolyte consisted of 1.2 M LiPF_6_ in ethylene carbonate (EC) and ethyl methyl carbonate (EMC) with a weight ratio of 3:7. All cell assembly procedures were conducted in an argon-filled glovebox to prevent moisture and oxygen contamination.

### 2.5 Electrochemical Evaluations

Galvanostatic charge–discharge tests were carried out in the voltage window of 3.5–4.95 V versus Li/Li^+^ using a Neware BTS-4000 battery testing system at room temperature. Prior to electrochemical evaluation, all cells were subjected to three formation cycles at 0.1 C to stabilize the electrode–electrolyte interface, assuming a theoretical capacity of 147 mAh/g at 1 C. Rate capability was subsequently assessed by charging at 0.2 C and discharging at progressively increased current densities of 0.2, 0.5, 1, 2, 5, and 10 C, with three cycles for each rate, followed by a recovery test at 0.2 C. Long-term cycling tests were conducted at 0.5 C for both charge and discharge after formation.

Electrochemical impedance spectroscopy measurements were conducted using a Gamry Interface 1010E workstation with a perturbation amplitude of 5 mV over a frequency range from 10^6^ to 5 × 10^–2^ Hz. Prior to impedance analysis, cells were subjected to three formation cycles and charged to 50% state of charge. Cyclic voltammetry was conducted in the voltage range of 3.5–5.0 V versus Li/Li^+^ at a scan rate of 0.1 mV/s using a Bio-Logic VSP potentiostat.

## 3 Results and Discussion

The introduction of Ca through a gradient-doping strategy can effectively modify the crystal structure of LNMO while preserving the primary spinel framework. Figure 1 shows the XRD patterns collected from the undoped and Ca gradient-doped samples. All diffraction peaks can be indexed based on the cubic spinel structure with *Fd*3̅*m* space group, and no reflections corresponding to the impurities or secondary Ca-containing phases are detected. This indicates that Ca gradient doping did not alter the primary crystal structure of LNMO, even at the highest doping level studied here (Ca gr 0.1 LNMO). Furthermore, the absence of superstructure reflections associated with the ordered *P*4_3_32 phase indicates that cation disorder is retained (Figure 1a).^25^ Closer examination of the diffraction peak positions, supported by the Rietveld refinement results (Table 1), reveals a shift of the (400) reflection toward a lower angle as doping level increases from 0.01 to 0.1 (Figure 1b), confirming the successful substitution of larger Ca^2+^ ions (1.00 Å, CN = 6) for smaller Ni^2+^ (0.69 Å) or Mn^4+^ (0.53 Å) in LNMO sample. The Ca gr 0.01 LNMO cathode has a (400) peak located at 44.31°, and it shifts to 44.22° for the Ca gr 0.1 sample, corresponding to an increase of the lattice parameter from 8.168 to 8.175 Å (Table 1). A linear fit across the gradient-doped series confirms this relationship, yielding a slope of ∼0.00068 Å per 0.01 Ca gradient with *R* ^2^ ≈ 0.996 (Figure 1c). This is consistent with Vegard’s law, which states that the lattice parameter of a solid solution varies approximately linearly with composition.^26^ Notably, the overall expansion remains modest in all gradient-doped samples, indicating that Ca incorporation is primarily surface-enriched and imposes little bulk strain (Figure 1c). In contrast, the uniformly doped samples exhibit a much higher slope of ∼0.00261 Å per 0.01 Ca, indicating a more extensive bulk substitution of Ni/Mn sites by Ca and consequently greater volumetric distortion of the spinel framework.

**Table 1:** Rietveld Refinement Data of the XRD Pattern for Ca Gradient-Doped LNMO Cathodes

LNMO cathodes	a (Å)	cell volume (Å^3^)	(111) fwhm (°)	2θ of (400) (°)
undoped	8.168	544.938	0.081	44.32
Ca gr 0.01	8.169	545.138	0.083	44.31
Ca gr 0.03	8.170	545.339	0.081	44.30
Ca gr 0.05	8.171	545.539	0.081	44.28
Ca gr 0.07	8.173	545.939	0.081	44.26
Ca gr 0.1	8.175	546.340	0.083	44.22

**Figure 1:**
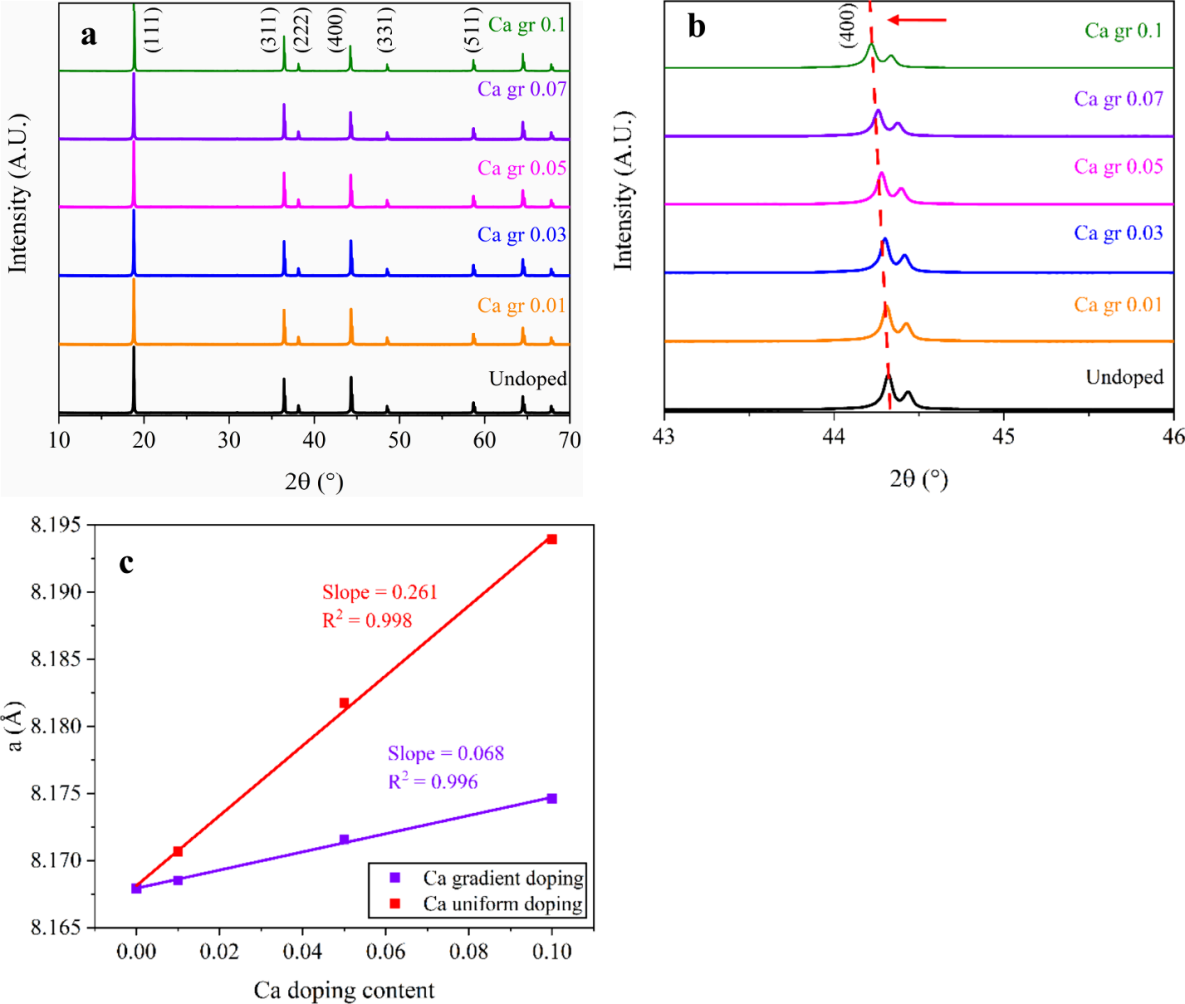
XRD patterns (a) of LNMO cathode materials synthesized with different Ca gradient doping levels (0.01–0.1). Enlarged view (b) of the 43–46° 2θ region highlighting the (400) reflection to show the peak shift with doping. Lattice parameter (c) from XRD Rietveld refinement as a function of Ca doping content for uniform and gradient-doped LNMO sample.

Gradient doping has a profound effect on the surface facet stabilization. This is supported by the analysis on the evolution of the intensity ratio of (400)/(111) peak (*I*_(400)_/*I*_(111)_) as a function of doping level (Table S1). As the gradient doping level increases, the ratio of *I*_(400)_/*I*_(111)_ linearly decreases from 0.54 (Ca gr 0.01 LNMO) to 0.38 (Ca gr 0.1 LNMO) (Table S1). Since (100) facets are thermodynamically less stable and associated with higher surface energies, while (111) facets are more stable with denser atomic packing, the reduced *I*_(400)_/*I*_(111)_ ratio indicates preferential growth of (111) surfaces in gradient-doped LNMO.^4^ Peak broadening also highlights differences between gradient and uniform doping (Table 1). The full width at half-maximum (fwhm) values of the (111) remain almost constant across gradient-doped samples (∼0.081–0.083°), indicating minimal impact on crystallite size. This suggests that gradient Ca primarily influences surficial facet stabilization without inducing substantial bulk structural distortions. Overall, XRD analysis confirms that Ca gradient doping stabilizes LNMO through surface enrichment of dopants and minimal bulk structure distortion. These structural features are beneficial factors for achieving long cycle life of LNMO cathodes through the enhanced surface stability while preserving bulk disorder.

The effect of Ca gradient doping on LNMO particle morphology can be further corroborated by the SEM observations (Figure 2). In the absence of Ca dopant, the undoped LNMO sample (Figure 2a) is composed of polyhedral particles primarily showing truncated octahedral morphologies with mixed (100) and (111) surface facets, along with a few irregular particles. The particle size is distributed over a relatively wide range of 0.8–3.7 μm, with an average of ∼1.8 μm. When a low Ca gradient level (0.01–0.03) is introduced (Figure 2b,c), the overall morphology remains truncated octahedral, but the particle edges become sharper, indicating a slight reduction of (100) facet exposure. With further increase of the Ca gradient levels (0.05–0.07) (Figure 2d,e), the particles evolve into well-defined octahedra with sharp edges, suggesting preferential growth of the thermodynamically stable (111) facets. Under these conditions, the size distribution becomes more uniform (1–2 μm) with an average of ∼1.2 μm. When the Ca gradient level is further increased to 0.1 (Figure 2f), the particles remain sharply faceted octahedra rather than reverting to truncated shapes, and the particle size decreases slightly to ∼0.9–1.2 μm with a relatively narrow distribution. These morphological changes are consistent with the XRD results (Table S1), which show that the *I*(400)/*I*(111) ratio decreases from the undoped sample to Ca gr 0.1, confirming suppressed (100) facet contribution at higher gradient levels. These observations collectively suggest that Ca gradient doping promotes preferential stabilization of the (111) surface planes. This is likely due to Ca enrichment near the particle surface, which modifies surface energies during crystal growth and favors the preferential growth of thermodynamically stable, densely packed (111) facets.^27^ Therefore, Ca gradient doping is expected to enhance surface stability, mitigate side reactions, and ultimately improve the electrochemical performance of Ca gradient-doped LNMO cathodes.

**Figure 2:**
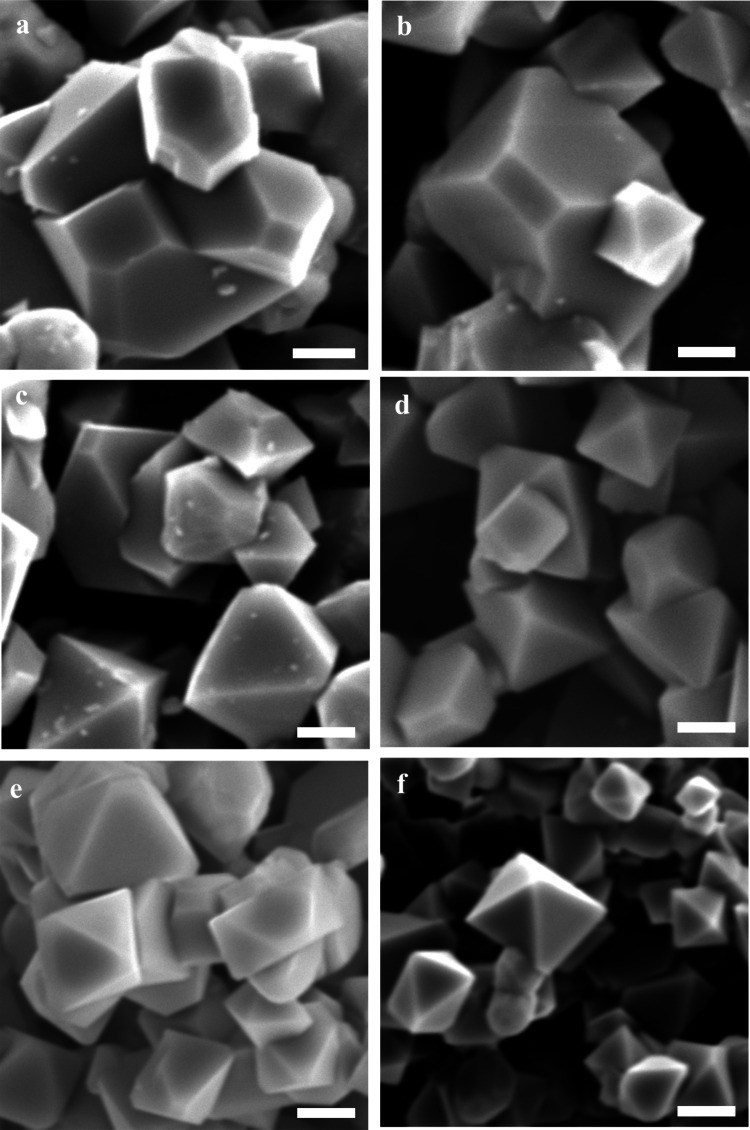
SEM micrographs of undoped LNMO (a), Ca gr 0.01 (b), Ca gr 0.03 (c), Ca gr 0.05 (d), Ca gr 0.07 (e), and Ca gr 0.1 (f). The scale bars represent 1 μm.

The effect of Ca gradient doping on the microstructure of the LNMO cathode materials is revealed by TEM observations. As shown in Figure 3a, undoped LNMO exhibits continuous and well-defined lattice fringes in the high-resolution TEM (HRTEM) images, indicative of single-crystalline particles with excellent structural integrity. The measured lattice spacing corresponds to the {311} planes of the cubic spinel structure (*Fd*3̅*m*), which is in good agreement with the XRD results. The corresponding selected-area electron diffraction (SAED) pattern displays sharp and discrete diffraction spots, confirming the XRD results (Figure 1) that the undoped LNMO crystallizes as a well-ordered single crystal without secondary phases. Similar features are observed for the Ca gr 0.05 and 0.1 LNMO samples (Figure 3d,f). Notably, no superlattice reflections are observed for all samples (Figure 3b,d,f), which confirms that the disordered spinel structure is preserved even after high Ca gradient incorporation.^28^ It has been reported that a high dopant concentration could perturb the LNMO spinel lattice and modify the bulk Ni/Mn ordering of LNMO.^21,29^ However, such dopant-induced Ni/Mn ordering is not observed in Ca gradient-doped samples, even at the highest Ca content of Ca gr 0.1. In contrast, the Ca uniform 0.1 sample exhibits clear superlattice reflections, indicating the formation of long-range Ni/Mn cation ordering, whereas the Ca gr 0.1 maintains a disordered structure without observable superlattice reflections (Figure 3f and Figure S1). Therefore, the gradient-doping approach prevents excessive lattice distortion induced by a high Ca^2+^ dopant and maintains the fully disordered spinel phase.

**Figure 3:**
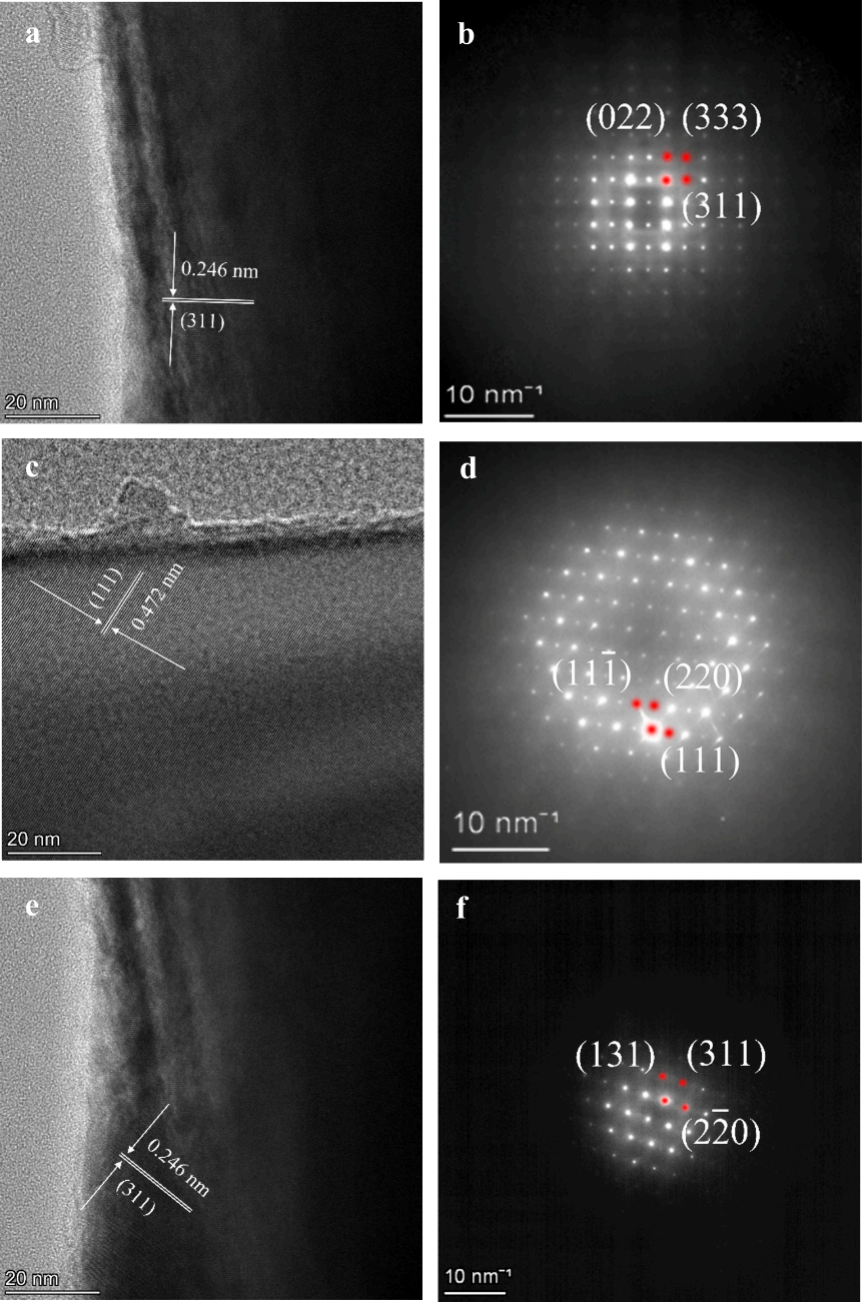
TEM images (a, c, e) and the corresponding electron diffraction (ED) patterns (b, d, f) of undoped LNMO, Ca gr 0.05, and Ca gr 0.1, respectively.

The distinct difference in cation ordering between the uniform and gradient-doped samples can be fundamentally rationalized from the lattice strain and dopant site-occupation energies. As reported in our previous structural analysis of uniformly Ca-doped LNMO,^21^ substituting native Ni^2+^ (0.69 Å) or Mn^4+^ (0.53 Å) with significantly larger Ca^2+^ ions (1.00 Å) throughout the bulk introduces severe global lattice strain. From a thermodynamic perspective, forcing highly mismatched dopant ions into bulk octahedral sites drastically increases the local defect formation energy and the ionic site occupation penalty. In a disordered *Fd*3̅*m* lattice, the random distribution of Ni and Mn creates a highly frustrated local environment that cannot efficiently accommodate this massive isotropic expansion. Conversely, prior density functional theory (DFT) calculations on LNMO spinels demonstrate that the periodic, symmetric framework of the ordered *P*4_3_32 phase allows for cooperative lattice relaxation, effectively minimizing the elastic strain energy and defect formation penalties.^30–32^ Accordingly, homogeneous Ca incorporation provides a thermodynamic driving force for transition-metal rearrangement, thereby relieving lattice distortion and promoting the long-range Ni/Mn ordering observed in the uniformly doped sample (Figure S1).

By contrast, the gradient-doping strategy confines Ca^2+^ predominantly to surface-enriched regions. The crystal surface, characterized by reduced atomic coordination and enhanced structural flexibility, provides a more favorable environment for accommodating dopant-induced lattice distortion. This reduces the energetic penalty of Ca incorporation and suppresses strain propagation into the bulk.^33–35^ Consequently, the bulk is less affected by extensive dopant-induced distortion, and the associated strain energy and defect formation energies are expected to remain comparatively low. With a reduced thermodynamic driving force for cation rearrangement, the gradient-doped sample retains the disordered *Fd*3̅*m* phase within the bulk. This strain-relief and energy-minimization mechanism is highly consistent with recent computational studies regarding dopant-induced lattice evolution and structural phase stability in advanced multi-ion cathode materials.^36,37^

To further verify the distribution of Ca in gradient-doped LNMO, scanning electron microscopy–energy-dispersive X-ray spectroscopy (SEM-EDS) line profiles and electron microprobe analysis (EPMA) were conducted. Both SEM–EDS and EPMA line scans of the Ca gradient-doped samples exhibit a slight enrichment of Ca near the particle surface relative to the particle center (Figures 4 and 5). This observation suggests that gradient doping preferentially enriches Ca in the outer regions of the spinel particles during synthesis. It has been well established that dopant ionic size can strongly influence lattice strain and, consequently, the structural ordering behavior of the host framework of LNMO materials.^29,38,39^ Due to the relatively large ionic radius of Ca^2+^(1.00 Å) compared to Ni (0.69 Å) or Mn (0.53 Å), Ca^2+^ incorporation at a high doping level (e.g., Ca gr 0.1) is expected to impose substantial lattice strain in LNMO. To mitigate such lattice strain, the surface-enriched Ca distribution enables the incorporation of the relatively large Ca^2+^ dopants while still maintaining a disordered *Fd*3̅*m* bulk (Figure 3b,d,f). Overall, these results demonstrate that gradient doping is an effective strategy to achieve higher Ca incorporation while preserving bulk disorder and providing surface stabilization.

**Figure 4:**
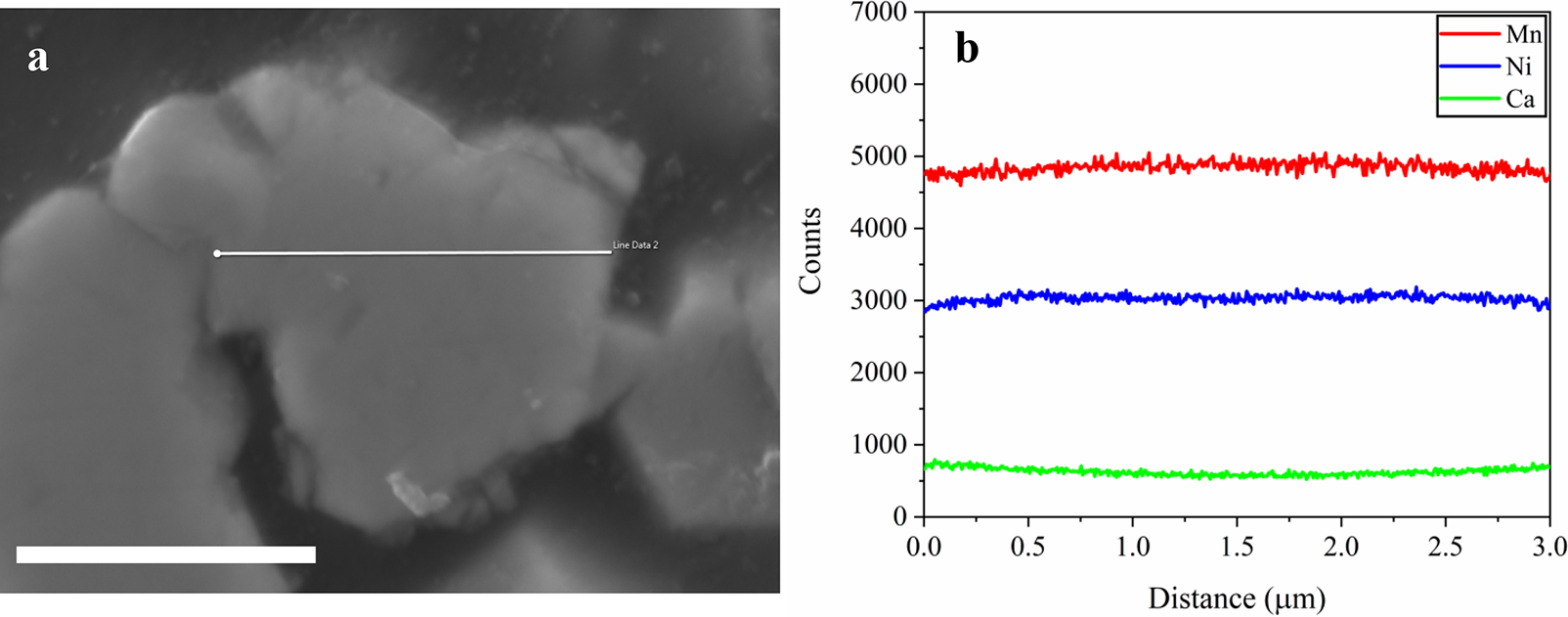
Cross-sectional SEM image (a) EDS line profiles for Ni, Mn, and Ca (b) along the white line marked in SEM image of an Ca 0.05 gradient-doped LNMO particle. The scale bar represents 2.5 μm.

**Figure 5:**
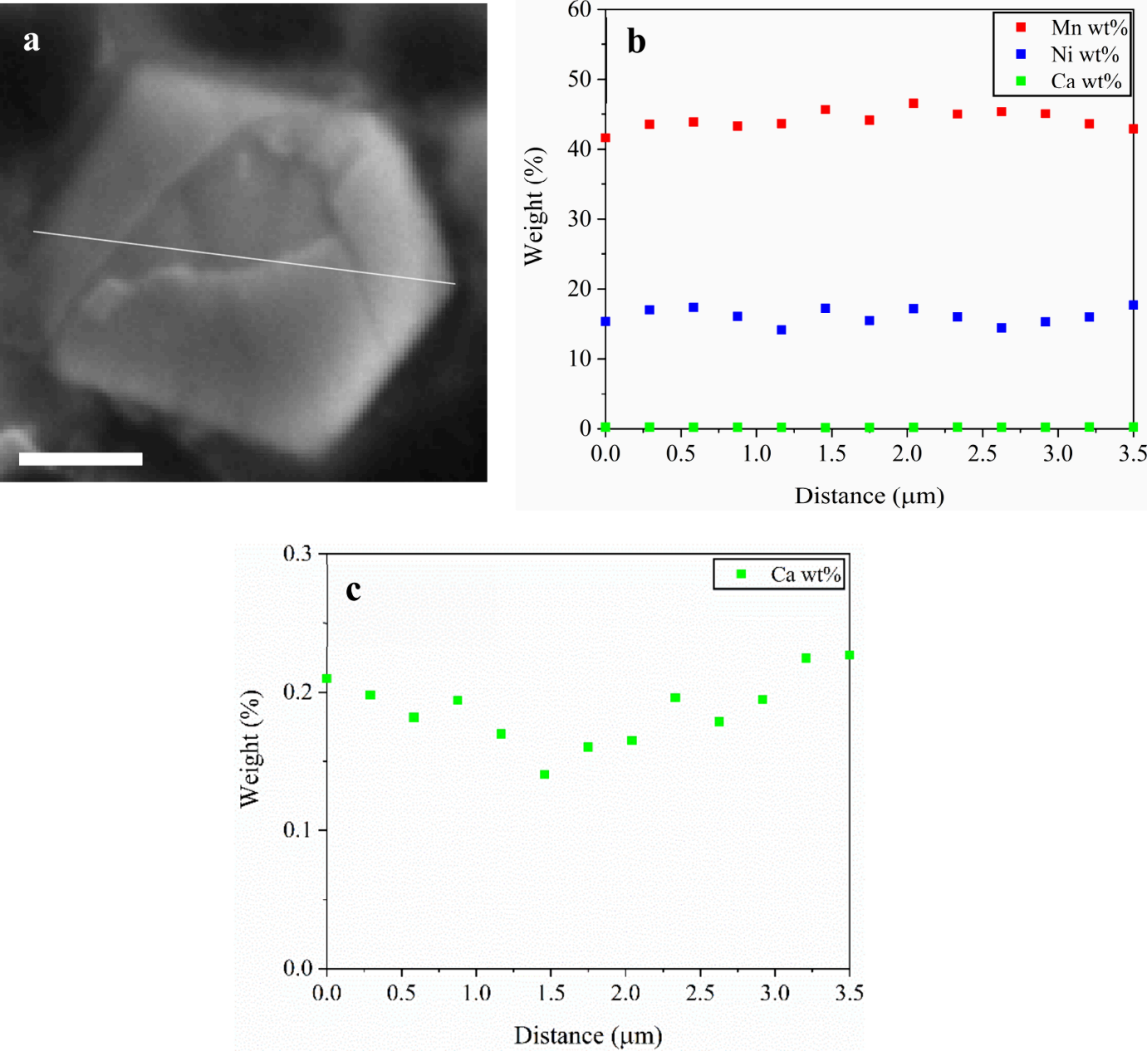
Cross-sectional EPMA image (a) of Ca 0.05 gradient-doped LNMO particle, full-scale weight-percent profiles (b) for Mn, Ni, and Ca, and enlarged figure (c) to highlight surface enrichment of Ca at the particle edges. The EPMA line scan was collected along the white line marked in the image. The scale bar represents 1 μm.

The structural and morphological evolution under Ca gradient doping is governed by the interplay of lattice strain, surface energies, and facet growth kinetics. Specifically, the preferential stabilization of (111) facets of both Ca uniform and gradient-doped LNMO can be rationalized by considering the combined effects of surface energies and growth kinetics. In the spinel structure, the (111) planes are close-packed oxygen-terminated surfaces, exhibiting the highest atomic density and strongest metal–oxygen coordination, while the (100) planes are relatively open, metal-rich, and contain a higher fraction of unsaturated bonds.^40^ According to crystal growth energy models, close-packed, low-energy planes such as (111) contain fewer unsatisfied bonds and higher nucleation barriers, which together slow their normal growth and cause them to remain as the dominant exposed facets in the final morphology.^41–45^ The incorporation of Ca further reduces the energy of (111) planes. Due to its large ionic radius and strong affinity for oxygen, Ca^2+^ preferentially segregates to oxygen-rich (111) planes, where the strong Ca–O bonds further lower the surface energy of (111) relative to (100). As a result, (111) planes grow more slowly after Ca doping, and remain preferentially exposed, leading to (111) dominated octahedra in Ca gradient-doped LNMO. Similar crystal facet growth mechanisms have been reported extensively in previous studies.^11,46,47^ Beyond (111) facet control, Ca gradient doping also improves interfacial stability through another mechanism at the particle surface. Studies indicate that Mn dissolution is exacerbated by surface oxygen vacancies and high Mn^3+^ content, particularly on reactive facets.^48–50^ By enriching Ca near the surface, these vacancies are less likely to form due to the increased energy barrier for oxygen release by the formation of strong Ca–O bonds. At the same time, fewer Mn^3+^ species near the interface could reduce disproportionation reaction (2Mn^3+^ → Mn^2+^ + Mn^4+^) and Mn^2+^ dissolution into the electrolyte. The gradient profile thus allows the use of higher overall Ca dopant without triggering Ni/Mn ordering, which is expected at high doping levels^29,38^ and is confirmed in this study (Figure S1), while still providing a chemically resilient outer shell. In summary, it is proposed that Ca gradient doping stabilizes (111) facets even at elevated Ca contents and suppresses Mn dissolution, yielding a (111) rich surface over a disordered bulk. These structural and interfacial benefits are expected to enhance the electrochemical performance of Ca gradient-doped LNMO cathodes.

The advantages of Ca gradient doping can be validated through the improved performance of LIBs with LNMO cathode materials. Figure 6a shows the third-cycle formation charge–discharge profiles of undoped LNMO, uniformly doped Ca 0.05, and gradient-doped LNMO samples with different Ca levels. The Ca uniform 0.05 sample was selected as a reference based on screening experiments of uniformly doped LNMO, which identified this composition as the optimal uniform doping condition. All LNMO cathodes exhibit three characteristic voltage plateaus: a low-voltage plateau around 4.0 V associated with the Mn^3+^/Mn^4+^ redox couple, and two high-voltage plateaus near 4.7 V corresponding to the sequential Ni^2+^/Ni^3+^ and Ni^3+^/Ni^4+^ redox reactions observed during charge and discharge. The impact of Ca gradient doping on the electrochemical properties of LNMO cathodes can be clearly seen from the discharge capacity results. The undoped and Ca gr 0.01 samples exhibit the highest first-cycle capacities of ∼131 mAh/g, while higher-level gradient doping (0.05–0.10 gr) lowers the capacity to ∼125–128 mAh/g. Although this represents a modest reduction in total capacity, it is consistent with the capacity trade-off commonly reported in the literature, where elemental doping slightly sacrifices initial capacity in exchange for improved cycling stability. This slight capacity reduction is consistent with XRD and SEM results of slight lattice expansion and well-defined octahedral morphologies with dominant (111) exposure at higher gradient levels. Such structural features are known to slightly reduce the accessible Li inventory at the surface while simultaneously enhancing the bulk stability. Despite the modest capacity decrease relative to undoped LNMO, Figure 6b shows that gradient-doped LNMO still delivers higher third-cycle formation capacities than uniformly doped samples at the same Ca content for every doping level. This indicates that the gradient distribution preserves more of the bulk active site, thereby enabling higher utilization of bulk capacity during cycling.

**Figure 6:**
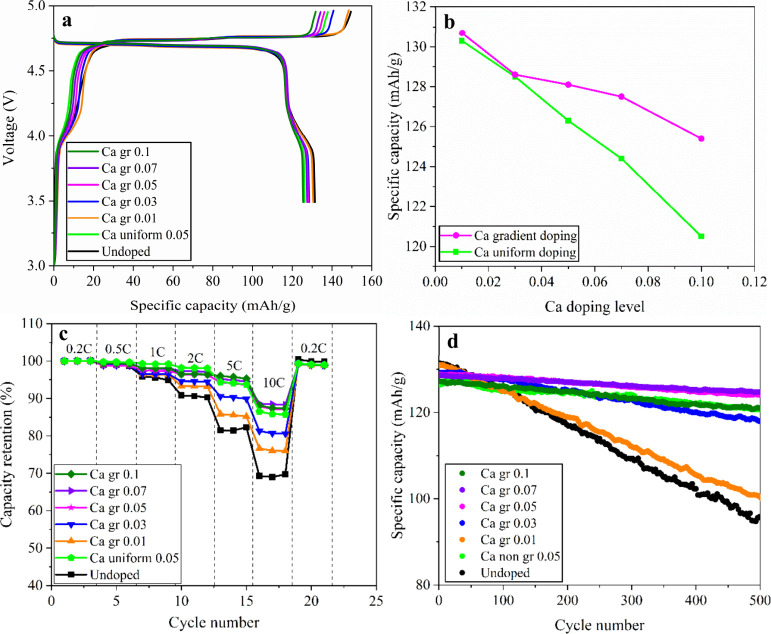
Electrochemical performance of LNMO cathodes with various Ca doping strategies. (a) Third-cycle formation charge–discharge voltage profiles of undoped, Ca uniform-doped, and Ca gradient-doped LNMO cathodes. (b) Comparison of third-cycle formation discharge capacities of Ca uniform-doped and Ca gradient-doped LNMO cathodes at different Ca doping levels. (c) Rate capability and (d) cycling performance of Ca-doped LNMO cathodes measured at 25 °C and 0.5 C.

Polarization analysis further underscores the beneficial role of Ca gradient doping (Table S2). The baseline undoped sample suffers from the largest polarization (0.0204 V), whereas Ca gr samples show significantly lower values (0.0102 V for Ca gr 0.07), demonstrating enhanced charge-transfer kinetics and reduced interfacial resistance. These results directly correlate with the structural characteristics of LNMO that uniform Ca doping induces bulk strain that hinders Li^+^ transport, whereas gradient doping simultaneously preserves the disordered *Fd*3̅*m* bulk phase and stabilizes (111) facets. This combination of bulk disorder and interfacial stabilization yields enhanced Li^+^ transport kinetics and suppressed surface resistance, thereby minimizing polarization.

In addition to polarization behavior, the influence of Ca gradient doping on the Mn^3+^/Mn^4+^ redox contribution can be clearly seen in the discharge profiles. The results are summarized in Table S3. The Mn^3+^/Mn^4+^ plateau length between 3.8 and 4.2 V (or capacity at low-voltage plateau) decreases progressively with Ca addition. While the undoped sample shows the largest Mn^3+^ fraction (∼9.2%, 12.1 mAh/g), gradient-doped samples exhibit a gradual decrease, reaching the lowest contribution at Ca gr 0.1 LNMO (∼6.1%). This suppression of the Mn^3+^ plateau is also observed in the Ca uniform 0.05 sample, which shows the lowest Mn^3+^ fraction of ∼6.1%. These results indicate that Ca incorporation, whether uniform or gradient, reduces the Mn dissolution and Jahn–Teller effect induced by Mn^3+^ species and strengthens the dominance of the Ni^2+^/Ni^4+^ redox process. The reduction of Mn^3+^ can be rationalized by two synergistic factors governed by defect chemistry and oxygen stoichiometry. For bulk incorporation, maintaining overall electroneutrality during the aliovalent substitution of higher-valent transition metals (Mn^4+^) by divalent Ca^2+^ promotes the oxidation of surrounding Mn^3+^ to Mn^4+^. For surface enrichment, the generation of Mn^3+^ is closely correlated with oxygen loss.^5^ Due to the significantly stronger Ca–O bonds compared to Mn–O or Ni–O,^20^ the enrichment of Ca^2+^ at the surface raises the thermodynamic energy barrier for oxygen release. By effectively suppressing the formation of surface oxygen vacancies, the concurrent reduction of Mn^4+^ to Mn^3+^ is largely mitigated. Gradient doping maximizes this benefit by enriching the oxygen-stabilizing Ca ions at the surface where oxygen loss typically initiates. Compared to uniform-doped LNMO (Ca uniform 0.05), gradient-doped samples exhibit stronger suppression of Mn^3+^ by surface-enriched Ca^2+^ that stabilizes the (111) facets. These low-energy planes are intrinsically less prone to oxygen release, thereby mitigating the formation of Mn^3+^ at the surface. In contrast, uniform Ca doping distributes Ca^2+^ across the entire lattice, which enhances Mn–O bonding and reduces Jahn–Teller distortion but also introduces lattice strain and cation ordering (Figure S1). Overall, these results suggest that Ca gradient doping suppresses Mn^3+^ primarily through interfacial stabilization, enabling improved electrochemical stability without altering the cation ordering of the bulk lattice.

This mechanistic distinction between interfacial and bulk effects is further reflected in the Coulombic efficiency (CE) of the samples (Table S2). The undoped LNMO exhibits the lowest CE of 87.9%, whereas gradient-doped samples at intermediate levels (0.05–0.07 gradient doping) reach ∼94–95%, with Ca gr 0.1 LNMO reaching the highest of 95.2%. By contrast, the uniformly doped Ca 0.05 sample shows a lower CE (91.6%) than the Ca gr 0.05 (94.1%) at a similar Ca content. The elevated CE in gradient-doped samples can be attributed to the Ca-rich surface shell, which stabilizes oxygen through strong Ca–O bonding and mitigates HF-driven Mn dissolution during the high-voltage plateau. Overall, Ca gr 0.05 LNMO achieves the optimal balance, with high discharge capacity, low polarization, reduced Mn^3+^ contribution, and high Coulombic efficiency. These improvements are mechanistically attributed to the establishment of a Ca-enriched surface that stabilizes (111) facets, preserves bulk disorder, and mitigates surface parasitic reactions via strong Ca–O bonding. Collectively, these structure–property correlations confirm that Ca gradient doping produces a surface–bulk structure that is favorable for long-term electrochemical stability in LNMO cathodes.

The advantages of gradient doping can also be found in the excellent rate performance of Ca gradient-doped LNMO cathodes. The capacity retention can be significantly affected by the Ca gradient doping effect (Figure 6c, Figure S2, and Tables S4 and S5). At a low rate of 0.2 C, all samples deliver high discharge capacities (124–131 mAh/g), with undoped LNMO exhibiting the highest initial capacity (∼131.6 mAh/g) and Ca-doped samples showing slightly reduced values. As the rate increases, performance differences become increasingly pronounced. At 2 C, the undoped electrode decreases to ∼119.5 mAh/g (∼90.8%), whereas Ca-doped samples show effectively improved capacity retentions with ∼124.0 mAh/g (∼98.2%), ∼122.7 mAh/g (∼96.5%), and 122.5 mAh/g (∼97.3%) preserved in Ca uniform 0.05, Ca gr 0.05, and Ca gr 0.07 samples, respectively. The capacity gap becomes most pronounced at 10 C. The specific capacity delivered by undoped LNMO collapses to ∼91.1 mAh/g (∼69.3%), while Ca uniform 0.05 sample maintains ∼109.2 mAh/g (∼86.5%) at 10 C. Ca gr 0.05 delivers the best high-rate performance, sustaining capacities of ∼111.7 mAh/g (∼87.9%) at 10 C. Upon returning to 0.2 C, all samples recover close to their initial capacities, confirming structural reversibility during high rate cycling. Notably, the Ca gradient-doped 0.05 delivers higher discharge capacities than the Ca uniform 0.05 sample at high rates of 10 C, confirming that the gradient distribution of Ca promotes enhanced electrochemical kinetics and enables superior capacity retention at elevated rates compared to uniform doping.

The superior high-rate performance of Ca gradient-doped LNMO is corroborated by reduced voltage polarization (Figure S3 and Table S6). From 2 to 10 C, the polarization differences become more evident among samples. At 10 C, the undoped LNMO falls to 4.135 V at 50% DOD, giving a ΔV of 0.566 V, while the Ca 0.05 uniform-doped sample reduces this to 0.468 V. Gradient samples, however, further suppress polarization, with Ca gr 0.07 LNMO exhibiting the smallest voltage drop (0.458 V). The voltage drop data demonstrate that gradient Ca doping reduces polarization by simultaneously stabilizing the electrode surface and maintaining fast bulk diffusion. At low C-rates, polarization is primarily governed by interfacial processes, where Ca-enriched surfaces mitigate Mn dissolution, lowering interfacial resistance. This explains why Ca gradient-doped LNMO samples exhibit higher capacity retention than undoped LNMO across all tested rates. At elevated C-rates, the rate-limiting step shifts to Li^+^ transport through the spinel lattice, and Ca gradient doping preserves disordered bulk that provides interconnected three-dimensional Li^+^ diffusion pathways. The moderate Ca gradient-doped LNMO samples (Ca gr 0.05 & Ca gr 0.07) thus achieve the lowest polarization across all C-rates, while excessive enrichment (Ca gr 0.1) slightly increases ΔV due to partial overpassivation of the surface.

The cycling performance of LNMO electrodes further underscores the impact of Ca gradient doping on long-term structural stability (Figure 6d). At 25 °C and 0.5 C, the undoped LNMO delivers an initial discharge capacity of ∼131.4 mAh/g, but undergoes rapid fading, retaining only ∼95.5 mAh/g (∼72.6%) after 500 cycles. This rapid capacity decay in the undoped sample is highly characteristic of continuous Mn^3+^ disproportionation and subsequent dissolution. In contrast, the Ca-doped electrodes demonstrate markedly improved stability (Figure 6d). The optimized Ca uniform 0.05 sample achieves ∼121.0 mAh/g (94.4% retention) after 500 cycles. Gradient doping further stabilizes the LNMO cathode, with the Ca gr 0.05 LNMO retaining ∼124.1 mAh/g (∼96.3%) after 500 cycles. The beneficial effect of gradient doping is also evident under elevated-temperature cycling at 55 °C. As shown in Figure S4, the Ca gr 0.05 LNMO consistently delivers the highest capacity among all samples over 200 cycles, maintaining ∼103.7 mAh/g after 200 cycles, which is substantially higher than Ca 0.1 (∼91.7 mAh/g), Ca gr 0.01 (∼77.4 mAh/g), and undoped LNMO (∼74.0 mAh/g). This result indicates that the Ca gradient structure not only improves room temperature cycling durability, but also more effectively preserves structural integrity and interfacial stability at elevated temperatures, where transition-metal dissolution and electrolyte side reactions are typically accelerated.^51^ While direct quantitative analysis of the electrolyte would be required to quantitatively measure the exact concentration of dissolved transition metals, the comprehensive electrochemical data strongly support the suppression of Mn dissolution in the gradient-doped samples. As shown in Figure S5, the Mn^3+^ plateau at ∼4.0 V remains nearly constant throughout 500 cycles for the Ca gr 0.05 LNMO, indicating that no significant new Mn^3+^ species are generated during prolonged cycling. Furthermore, the Ca gr 0.05 LNMO maintains a higher Coulombic efficiency (∼91.7%) than the Ca 0.05 uniform-doped (∼90.1%) and undoped samples (∼86.5%) after 500 cycles (Figure S6). Since Mn dissolution is directly correlated with parasitic electrolyte oxidation and CEI thickening, the highly stable CE and the stabilized Mn^3+^ plateau provide robust electrochemical evidence of a protected electrode–electrolyte interface. This indicates that the Ca-enriched (111) surfaces effectively shield the lattice from HF attack, thereby mitigating transition-metal dissolution over prolonged cycling.^52–54^

To investigate the underlying mechanisms of the superior rate capability of Ca gradient-doped LNMO, electrochemical impedance spectroscopy (EIS) was performed, and the representative Nyquist spectra are shown in Figure 7. Each spectrum features a high-frequency depressed semicircle (*R*_CEI_), a midfrequency depressed semicircle (*R*_ct_), and a low-frequency tail associated with Warburg diffusion of Li^+^, consistent with a Randles-type response in LIBs. The undoped LNMO exhibits the largest semicircles, evidencing sluggish interfacial charge transfer and a comparatively resistive CEI (Figure 7a). Introducing uniform Ca at 0.05 markedly reduces both semicircles, where modest lattice expansion and reduced particle size facilitated charge transfer. Gradient doping further enhances interfacial kinetics, with Ca gr 0.05 LNMO exhibiting a smaller *R*_CEI_ and *R*_CT_ than Ca uniform 0.05 (Figure 7a). Among the gradient-doped samples, Ca gr 0.05 & Ca gr 0.07 exhibit the smallest *R*_CEI_ and *R*_CT_ among all compositions (Figure 7b). By contrast, the Ca 0.01 gradient-doped sample shows only a slight decrease of semicircles relative to undoped LNMO, and Ca gr 0.1 LNMO shows an increase in interfacial impedance at excessive surface Ca enrichment (Figure 7b). The diffusion coefficients calculated from the low-frequency region also follow the same ordering (Figure 7c): Ca gr 0.07 (1.4 × 10^–12^ cm^2^/s) > Ca gr 0.05 (1.3 × 10^–12^ cm^2^/s) > Ca gr 0.1 (1.14 × 10^–12^ cm^2^/s) > Ca uniform 0.05 (1.13 × 10^–12^ cm^2^ /s) > Ca gr 0.01 (3.3 × 10^–13^ cm^2^/s) > undoped (2.27 × 10^–13^ cm^2^ s^–1^). These trends are consistent with a Ca-enriched surface that stabilizes low-energy (111) facets, suppresses Mn^3+^-driven parasitic reactions, and limits CEI thickening, while preserving bulk *Fd*3̅*m* disorder and rapid Li^+^ diffusion at moderate gradient doping levels. In summary, EIS results demonstrate that gradient Ca doping further lowers interfacial resistance and enhances Li^+^ diffusion compared to uniform doping, consistent with its superior rate performance.

**Figure 7:**
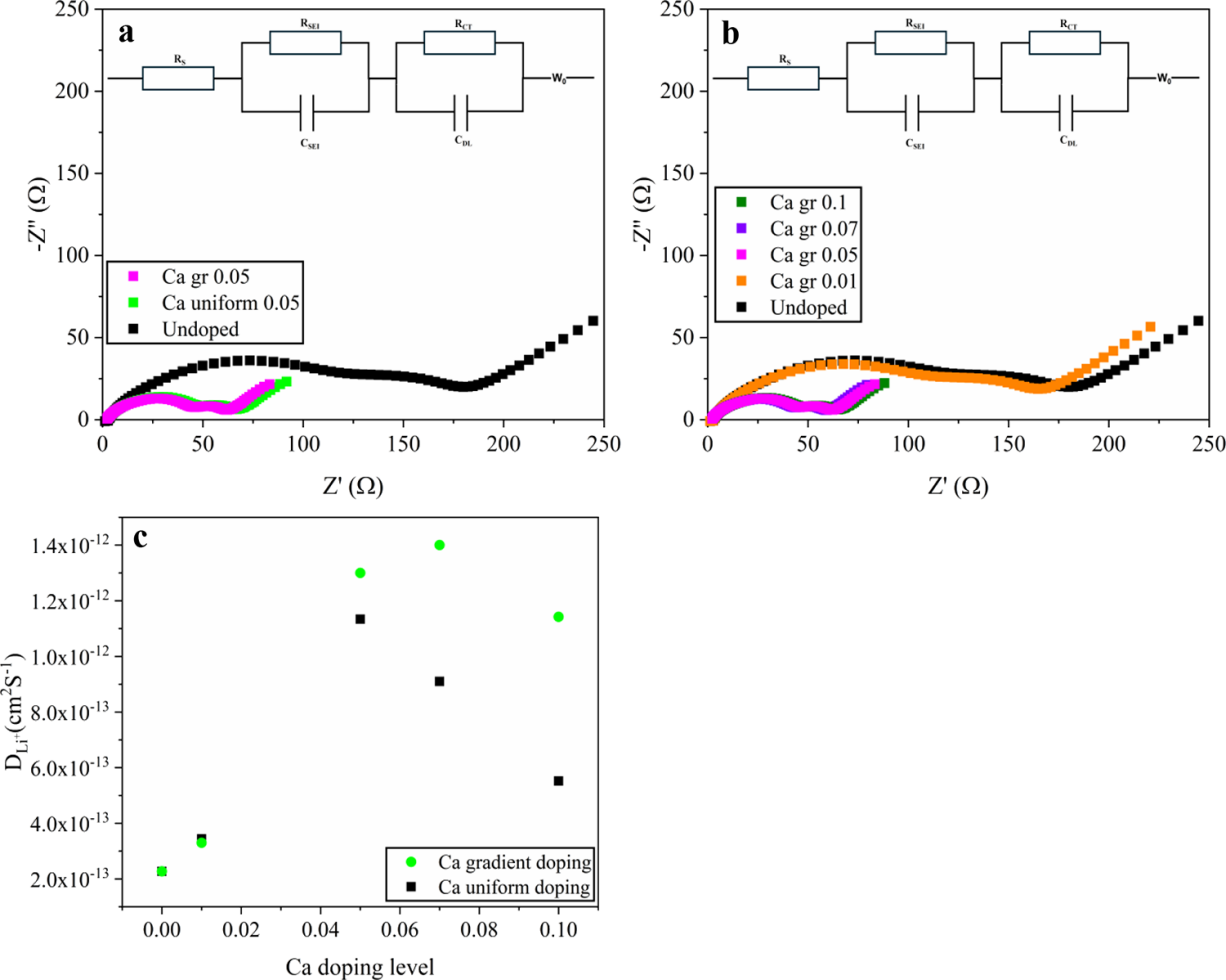
Nyquist plots of Ca 0.05 uniform- and gradient-doped LNMO cathodes (a), gradient-doped LNMO cathodes with various Ca doping levels (b), and lithium-ion diffusion coefficients (*D*_Li_^+^) as a function of Ca doping level for uniform and gradient doping (c). Before EIS measurements, all cells underwent three formation cycles, and then were charged to 50% SOC. The insets show the equivalent circuit for data fitting.

In addition, the direct correlation between bulk cation disorder and enhanced transport kinetics is mostly evident when comparing the high-doping-level samples. While the Ca uniform 0.1 sample exhibits superlattice reflections indicative of a transition to the ordered phase (Figure S1), the Ca gr 0.1 sample retains the disordered symmetry (Figure 3f). The preservation of disordered bulk structure results in a significantly higher lithium-ion diffusion coefficient than that of Ca uniform 0.1 (Figure 7c). This kinetic advantage is further reflected in the high-rate performance, where the disordered Ca gr 0.1 electrode delivers ∼109 mAh/g at 10 C (Figure S2). These results suggest that the gradient doping profile effectively suppresses dopant-induced ordering and contributes to the superior rate capability of gradient-doped LNMO electrodes.

Cyclic voltammetry (CV) provides direct insight into how Ca gradient doping influences the redox behavior and reaction kinetics of LNMO (Figure 8). All electrodes display the three characteristic redox processes of the LNMO spinel, a weak peak at ∼4.0 V corresponding to the Mn^3+^/Mn^4+^ couple, and two strong peaks between 4.7–4.9 V associated with the Ni^2+^/Ni^3+^ and Ni^3+^/Ni^4+^ redox reactions (Figure 8a). The anodic and cathodic peak separations are summarized in Table S7. The undoped electrode shows the largest anodic–cathodic separations for both Ni couples (Δ*V* = 0.272 V for Ni^2+^/Ni^3+^ and 0.238 V for Ni^3+^/Ni^4+^), indicating sluggish Li^+^ kinetics. With Ca 0.05 uniform doping, modest improvements are observed as the separations decrease to 0.254 and 0.23 V, respectively. Gradient Ca doping further enhances electrode reversibility. At the optimal gradient level of Ca gr 0.05, the Ni peak separations diminish to 0.234 V (Ni^2+^/Ni^3+^) and 0.2 V (Ni^3+^/Ni^4+^), the smallest among all samples. In contrast, at higher gradient doping (Ca gr 0.1 LNMO), the separations become larger again (0.26 and 0.242 V), indicating increased interfacial resistance. These results show that surface Ca enrichment facilitates Ni redox reversibility while excessive Ca enrichment produces a thicker interfacial layer that impedes Li^+^ transport. Notably, the two high-voltage Ni peaks remain distinctly resolved with no peak merging in all samples, suggesting a predominantly disordered *Fd*3̅*m* structure rather than long-range Ni/Mn ordering.^40,51,55^ In addition to Ni redox behavior, CV results also reflect the evolution of the Mn^3+^/Mn^4+^ couple (Figure 8b). The undoped electrode exhibits the strongest Mn^3+^ redox peaks near 4.0 V, consistent with the high content of Mn^3+^. Both uniform and gradient Ca incorporation weaken this peak, with the suppression most pronounced at higher gradient levels (Ca gr 0.1 LNMO). This trend indicates that Ca incorporation, especially when surface-enriched, effectively mitigates Mn^3+^ redox activity and stabilizes the electrode–electrolyte interface. Collectively, these Mn^3+^ trends, along with the peak separation analysis, confirm that gradient Ca doping simultaneously suppresses parasitic Mn^3+^ redox activities and enhances Ni kinetics.

**Figure 8:**
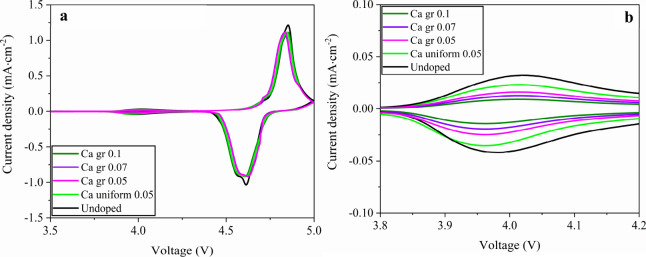
Full voltage range (a) and enlarged (b) cyclic voltammetry (CV) curves of LNMO cathodes. The CV curves in the enlarged voltage region from 3.8 to 4.2 V (b) highlight the Mn^3+^/Mn^4+^ redox peak.

The electrochemical performance of LNMO depends strongly on both its bulk structure and its surface characteristics. It has been well-recognized that the presence of reasonable Mn^3+^ content, high-active surface (111) facet, and high cation disorder can significantly improve the electrochemical performance of LNMO cathodes.^4,28,56^ TEM and SAED confirmed the preservation of disorder and absence of superlattice reflections for Ca gradient-doped LNMO samples, while EDS mapping confirmed that Ca is preferentially enriched at the particle surface in the gradient-doped LNMO cathodes. In addition, SEM observations revealed that Ca gradient doping promotes well-faceted octahedra enriched with (111) planes. These features enable Ca^2+^ gradient-doped LNMO cathodes, particularly at intermediate level (e.g., Ca gr 0.05 LNMO), with superior electrochemical performance through the synergistic effects of: (i) a disordered bulk structure that maintains solid-solution Li^+^ transport for fast Li^+^ diffusion kinetics, (ii) a Ca-enriched surface that stabilizes (111) facets and suppresses Mn dissolution and electrolyte decomposition, and (iii) optimized Mn^3+^ content that avoids both excessive Jahn–Teller distortion and ordering. These structure–property relationships explain the collective improvements in formation, rate, CV, cycling, and EIS results observed on Ca gradient-doped LNMO cathodes. Overall, a systematic evaluation of the electrochemical data, including the concentration-dependent initial capacity (Figure 6b), the evolution of CV peak separations (Table S7), and the calculated Li^+^ diffusion coefficients (Figure 7c), confirms that an optimal gradient doping level (Ca gr 0.05 to 0.07) provides the ideal balance between preserving active bulk capacity and enhancing interfacial charge-transfer kinetics.

To further demonstrate the performance advantages of the Ca gradient-doped LNMO developed in this work, a comparison with other gradient-doping and codoping strategies reported for LNMO in recent literature is summarized in Table 2. The optimized Ca gr 0.05 LNMO cathode demonstrates highly competitive initial capacity, superior high-rate capability (∼111.7 mAh/g at 10 C), and exceptional long-term cycling stability (96.3% capacity retention after 500 cycles at 0.5 C). Compared to other gradient-doped LNMO materials, such as Mg- and F gradient doping approaches, the Ca gradient strategy achieves a substantially longer cycle life and better capacity retention at extremely high rates. The significant enhancement in electrochemical performance can be attributed to the synergistic effects of surface and bulk stabilization. Specifically, the Ca-enriched surface promotes the preferential stabilization of low-energy (111) facets, thereby suppressing Mn dissolution and interfacial degradation, while the preservation of the disordered *Fd*3̅*m* bulk structure ensures highly reversible lithium storage and facilitates rapid Li^+^ diffusion kinetics.

**Table 2:** Comparison of the Electrochemical Performance of Ca Gradient-Doped LNMO with Other Gradient-Doped and Modified LNMO Cathodes Reported in the Literature

material	initial capacity	rate capability	cycling stability	references
Ca gr 0.05 LNMO	129.0 mAh/g (0.2 C)	111.7 mAh/g at 10 C	96.3% after 500 cycles (0.5 C)	this work
Mg gradient-doped LNMO	121.5 mAh/g (0.1 C)	91.4 mAh/g at 4.0 C	92.0% after 80 cycles (0.5 C)	^18^
F gradient-doped LNMO	124.7 mAh/g (1 C)	104.0 mAh/g at 10 C	92.4% after 300 cycles (1 C)	^9^
Mg^2+^/F^–^-codoped LNMO	144.0 mAh/g (0.5 C)	100.0 mAh/g at 10 C	86.2% after 400 cycles (5 C)	^10^
Ti-doped LNMO	127 mAh/g (0.2 C)	∼60 mAh/g at 5 C	90.0% after 300 cycles (1 C)	^61^
Ti/Zr co-doped LNMO	127.8 mAh/g (1 C)	∼60 mAh/g at 5 C	94.7% after 100 cycles (1 C)	^62^

Recent studies on advanced positive electrode materials further support the beneficial role of gradient compositional design in improving electrochemical performance. In Ni-rich layered oxides, Zeng et al. showed that structure and charge regulation through multicomponent modification can markedly improve cycling stability and rate capability by simultaneously stabilizing the bulk lattice and moderating interfacial reactivity, highlighting the importance of coupling structural robustness with controlled charge compensation behavior.^57^ More recently, a gradient microstructure design was reported to enhance the mechanochemical durability of single-crystalline Ni-rich layered oxides by constructing a surface-to-subsurface stability gradient, which suppressed intragranular cracking, reduced lattice shrinkage during phase transition, and delivered excellent long-term cycling stability even in a pouch-cell configuration.^58^ Similarly, Fan et al. demonstrated that fine-tuning the microstructure of Ni-rich cathodes can dissipate local strain accumulation and restrain lattice oxygen escape through strong metal–oxygen bonding. The modified electrodes exhibited improvement in both cycling durability and rate performance.^59^ In another study, phase-compatible surface engineering was employed to stabilize the near-surface lattice oxygen while maintaining rapid Li^+^ diffusion through heterogeneous-ion-induced lattice-spacing regulation, confirming that surface-protected/bulk active architectures are highly effective for high-energy cathodes.^60^ Although these studies primarily focus on Ni-rich layered oxides rather than spinel LNMO, they collectively establish a general principle that gradient or surface-enriched modification can further enhance the benefit of homogeneous bulk doping by increasing structural stabilization at the electrode–electrolyte interface, where degradation is most severe, while minimizing perturbation to the redox-active bulk and preserving intrinsic electrochemical kinetics. In the present Ca gradient-doped LNMO, this same design principle is realized through a Ca-enriched surface that stabilizes oxygen and suppresses Mn dissolution, along with a disordered *Fd*3̅*m* bulk that preserves fast Li^+^ transport. Therefore, the superior rate capability and long-term cycling stability of Ca gr 0.05 LNMO can be understood as a spinel-specific example of the broader performance advantages enabled by gradient doping in advanced cathode materials.

## 4 Conclusions

In this study, the gradient-doping strategy was used to synthesize LNMO cathodes with a stabilized surface, disordered structure in bulk, and appropriate Mn^3+^ content for high-performance LIBs. Combined with observations from TEM and SAED, the XRD analysis revealed that the gradient-doped samples retain the disordered *Fd*3̅*m* structure even at the highest Ca doping level (Ca gr 0.1 LNMO). SEM observations showed that Ca gradient doping promotes well-faceted octahedra enriched in (111) planes, and EDS mapping further confirmed that Ca is preferentially enriched on the particle surface in the gradient-doped LNMO cathodes.

As cathodes in LIBs, the Ca gradient-doped LNMO materials delivered formation capacities of ∼126–130 mAh/g, which are consistently higher than those of the uniform-doped samples at the same doping level, while substantially elevated Coulombic efficiency (88–95%) compared to both undoped (∼88%) and uniform-doped (∼92%) LNMO cathodes. Rate testing further highlighted the kinetic advantages of the gradient doping method with Ca gr 0.05 LNMO cathodes, maintaining ∼113 mAh/g at 10 C, while ∼92 and ∼110 mAh/g for undoped and Ca uniform 0.05 LNMO, respectively, demonstrating that gradient doping further enhances high-rate capacity compared with uniform doping. At the modest doping level, Ca gr 0.05 exhibits excellent cycling stability, retaining ∼124.1 mAh/g (∼96.3%) after 500 cycles. The analysis of CV, dQ/dV, and EIS revealed that the excellent electrochemical performance is attributed to the structural and morphological advantages of gradient-doped LNMO cathodes, in which the disordered bulk structure provides fast channels for fast Li^+^ diffusion, while a Ca-enriched surface stabilizes the (111) facets, minimizing the Mn dissolution into the electrolyte.

## Supplementary Material



## Notes

Additional electron diffraction patterns, rate capability as a function of current density, discharge voltage profiles at varied C-rates, high-temperature cycling performance, discharge voltage and differential capacity profiles at selected cycles, Coulombic efficiency plots, and summary tables of structural and electrochemical parameters (PDF)

## References

[1] Liang G., Peterson V. K., See K. W., Guo Z., Pang W. K. (2020). Developing
high-voltage spinel LiNi 0.5 Mn 1.5 O 4 cathodes for high-energy-density
lithium-ion batteries: current achievements and future prospects. Journal of Materials Chemistry A.

[2] Li W., Song B., Manthiram A. (2017). High-voltage
positive electrode materials
for lithium-ion batteries. Chem. Soc. Rev..

[3] Patoux S., Daniel L., Bourbon C., Lignier H., Pagano C., Le Cras F., Jouanneau S., Martinet S. (2009). High voltage spinel
oxides for Li-ion batteries: From the material research to the application. J. Power Sources.

[4] Yu X., Yu W. A., Manthiram A. (2021). Advances and
prospects of high-voltage
spinel cathodes for lithium-based batteries. Small Methods.

[5] Choi S., Feng W., Xia Y. (2024). Recent progress
of high voltage spinel
LiMn1. 5Ni0. 5O4 cathode material for lithium-ion battery: surface
modification, doping, electrolyte, and oxygen deficiency. ACS omega.

[6] Park N. R., Li Y., Yao W., Zhang M., Han B., Mejia C., Sayahpour B., Shimizu R., Bhamwala B., Dang B. (2024). Understanding
the role of lithium borate as the surface coating on high voltage
single crystal LiNi0. 5Mn1. 5O4. Adv. Funct.
Mater..

[7] Fu T., Lu D., Yao Z., Li Y., Luo C., Yang T., Liu S., Chen Y., Guo Q., Zheng C. (2023). Advances in modification
methods and the future prospects of high-voltage spinel LiNi 0.5 Mn
1.5 O 4—a review. J. Materi. Chem. A.

[8] Kim J. W., Kim D. H., Oh D. Y., Lee H., Kim J. H., Lee J. H., Jung Y. S. (2015). Surface chemistry
of LiNi0. 5Mn1.
5O4 particles coated by Al2O3 using atomic layer deposition for lithium-ion
batteries. J. Power Sources.

[9] Luo Y., Li H., Lu T., Zhang Y., Mao S. S., Liu Z., Wen W., Xie J., Yan L. (2017). Fluorine gradient-doped LiNi0. 5Mn1.
5O4 spinel with improved high voltage stability for Li-ion batteries. Electrochim. Acta.

[10] Wei A., Li W., Chang Q., Bai X., He R., Zhang L., Liu Z., Wang Y. (2019). Effect of
Mg2+/F– co-doping on electrochemical
performance of LiNi0. 5Mn1. 5O4 for 5 V lithium-ion batteries. Electrochim. Acta.

[11] Mao J., Dai K., Xuan M., Shao G., Qiao R., Yang W., Battaglia V. S., Liu G. (2016). Effect of chromium and niobium doping
on the morphology and electrochemical performance of high-voltage
spinel LiNi0. 5Mn1. 5O4 cathode material. ACS
Appl. Mater. Interfaces.

[12] Stüble P., Geßwein H., Indris S., Müller M., Binder J. R. (2022). On the electrochemical
properties of the Fe–Ti
doped LNMO material LiNi 0.5 Mn 1.37 Fe 0.1 Ti 0.03 O 3.95. Journal of Materials Chemistry A.

[13] Xiong, J. High Capacity and Long Cycle Life High Voltage Spinel Cathode Materials for Lithium-Ion Batteries. Western Michigan University, 2025.

[14] Hou P., Zhang H., Zi Z., Zhang L., Xu X. (2017). Core–shell
and concentration-gradient cathodes prepared via co-precipitation
reaction for advanced lithium-ion batteries. Journal of Materials Chemistry A.

[15] Liu T., Yu L., Lu J., Zhou T., Huang X., Cai Z., Dai A., Gim J., Ren Y., Xiao X. (2021). Rational design of
mechanically robust Ni-rich cathode materials via concentration gradient
strategy. Nat. Commun..

[16] Zeng X., Zhan C., Lu J., Amine K. (2018). Stabilization
of a
high-capacity and high-power nickel-based cathode for Li-ion batteries. Chem..

[17] Yu H., Cao Y., Chen L., Hu Y., Duan X., Dai S., Li C., Jiang H. (2021). Surface enrichment and diffusion
enabling gradient-doping
and coating of Ni-rich cathode toward Li-ion batteries. Nat. Commun..

[18] Liu M.-H., Huang H.-T., Lin C.-M., Chen J.-M., Liao S.-C. (2014). Mg gradient-doped
LiNi0. 5Mn1. 5O4 as the cathode material for Li-ion batteries. Electrochim. Acta.

[19] Shannon R. D. (1976). Revised
effective ionic radii and systematic studies of interatomic distances
in halides and chalcogenides. Foundations of
Crystallography.

[20] Luo, Y.-R. Comprehensive handbook of chemical bond energies; CRC press, 2007.

[21] Xiong J., Zhou B., Mathew K., Kornyo E., Zhang G., Lu W., Mei Z., Wu Q. (2026). Calcium-Doped High-Voltage Spinel
Cathode for Long Cycle Life Lithium-Ion Batteries. ACS Appl. Energy Mater..

[22] Pohorecki, R. ; Bałdyga, J. The effects of micromixing and the manner of reactor feeding on precipitation in stirred tank reactors. In Tenth International Symposium on Chemical Reaction Engineering, 1988; Elsevier: pp 1949–1954.

[23] Hu H.-Y., Li Y.-C., Zhu Y.-F., Liu H., Xiang W., Wang J.-Z., Xiao Y. (2025). Synthetic control guided by growth
mechanism insights enable tailored precursors for layered oxide cathodes. Chemical Science.

[24] Baḱldyga J., Podgórska W., Pohorecki R. (1995). Mixing-precipitation model with application
to double feed semibatch precipitation. Chem.
Eng. Sci..

[25] Manthiram A., Chemelewski K., Lee E.-S. (2014). A perspective on
the high-voltage
LiMn 1.5 Ni 0.5 O 4 spinel cathode for lithium-ion batteries. Energy Environ. Sci..

[26] Vegard L. (1921). Die konstitution
der mischkristalle und die raumfüllung der atome. Zeitschrift für Physik.

[27] Karim A., Fosse S., Persson K. A. (2013). Surface
structure and equilibrium
particle shape of the LiMn 2 O 4 spinel from first-principles calculations. Phys. Rev. B:Condens. Matter Mater. Phys..

[28] Xiao J., Chen X., Sushko P. V., Sushko M. L., Kovarik L., Feng J., Deng Z., Zheng J., Graff G. L., Nie Z. (2012). High-performance LiNi0.
5Mn1. 5O4 spinel controlled by Mn3+ concentration
and site disorder. Adv. Mater..

[29] Lee E.-S., Manthiram A. (2013). Influence
of doping on the cation ordering and charge–discharge
behavior of LiMn 1.5 Ni 0.5– x M x O 4 (M= Cr, Fe, Co, and
Ga) spinels between 5.0 and 2.0 V. Journal of
Materials Chemistry A.

[30] Cen J., Zhu B., Kavanagh S. R., Squires A. G., Scanlon D. O. (2023). Cation disorder
dominates the defect chemistry of high-voltage LiMn 1.5 Ni 0.5 O 4
(LMNO) spinel cathodes. Journal of Materials
Chemistry A.

[31] Shiiba H., Zettsu N., Nakayama M., Oishi S., Teshima K. (2015). Defect formation
energy in spinel LiNi0. 5Mn1. 5O4-δ using Ab initio DFT calculations. J. Phys. Chem. C.

[32] Lee E., Persson K. A. (2013). Solid-solution Li
intercalation as a function of cation
order/disorder in the high-voltage Li x Ni0. 5Mn1. 5O4 spinel. Chem. Mater..

[33] No̷rskov J. K., Bligaard T., Hvolbæk B., Abild-Pedersen F., Chorkendorff I., Christensen C. H. (2008). The nature
of the active site in
heterogeneous metal catalysis. Chem. Soc. Rev..

[34] Huang J., Liu H., Zhou N., An K., Meng Y. S., Luo J. (2017). Enhancing
the ion transport in LiMn1. 5Ni0. 5O4 by altering the particle Wulff
shape via anisotropic surface segregation. ACS
Appl. Mater. Interfaces.

[35] Ding H., Virkar A. V., Liu M., Liu F. (2013). Suppression of Sr surface
segregation in La 1– x Sr x Co 1– y Fe y O 3–
δ: a first principles study. Phys. Chem.
Chem. Phys..

[36] Fang Y., Zhao J., Su Y., Dong J., Lu Y., Li N., Wang H., Wu F., Chen L. (2024). Understanding of spinel
phases in lithium-rich cathode for high-energy-density lithium-ion
batteries: A review. Energy Mater. Adv..

[37] Wang Z., Li Y., Zhou Q., Li Q., Zhao R., Qiu Z., Zhang R., Sun Y., Wu F., Wu C., Bai Y. (2024). Multi-ion strategies toward advanced
rechargeable batteries: Materials,
properties, and prospects. Energy Mater. Adv..

[38] Lee J., Dupre N., Avdeev M., Kang B. (2017). Understanding the cation
ordering transition in high-voltage spinel LiNi0. 5Mn1. 5O4 by doping
Li instead of Ni. Sci. Rep..

[39] Strandbakke R., Wragg D. S., So̷rby M. H., Guzik M. N., Gunnæs A. E., Szpunar I., Wachowski S. L., Balaguer M., Carvalho P. A., Mielewczyk-Gryń A. (2022). Structural
properties of mixed conductor
Ba 1– x Gd 1– y La x+ y Co 2 O 6– δ. Dalton Trans..

[40] Li Z.-Q., Liu Y.-F., Liu H.-X., Zhu Y.-F., Wang J., Zhang M., Qiu L., Guo X.-D., Chou S.-L., Xiao Y. (2024). Kinetically controlled synthesis of low-strain disordered micro–nano
high voltage spinel cathodes with exposed {111} facets. Chemical Science.

[41] Woensdregt C. F. (1993). Hartman–Perdok
theory: influence of crystal structure and crystalline interface on
crystal growth. Faraday Discuss..

[42] Hartman P., Perdok W. (1955). On the relations between
structure and morphology of
crystals. III. Acta Crystallogr..

[43] Hartman P., Bennema P. (1980). The attachment energy
as a habit controlling factor:
I. Theoretical considerations. J. Cryst. Growth.

[44] Burton W.-K., Cabrera N. t., Frank F. (1951). The growth of crystals and the equilibrium
structure of their surfaces. Philosophical Transactions
of the Royal Society of London. Series A, Mathematical and Physical
Sciences.

[45] Uwaha M. (2016). Introduction
to the BCF theory. Progress in Crystal Growth
and Characterization of Materials.

[46] Liu H., Kloepsch R., Wang J., Winter M., Li J. (2015). Truncated
octahedral LiNi0. 5Mn1. 5O4 cathode material for ultralong-life lithium-ion
battery: Positive (100) surfaces in high-voltage spinel system. J. Power Sources.

[47] Chemelewski K. R., Shin D. W., Li W., Manthiram A. (2013). Octahedral
and truncated high-voltage spinel cathodes: the role of morphology
and surface planes in electrochemical properties. Journal of Materials Chemistry A.

[48] Nong J., Zhao X., Liang F., Jia S., Zou Z. (2025). Surface Oxygen
Vacancy Modulation of Nanostructured Li-Rich Mn-Based Oxides for Lithium-Ion
Batteries. Materials.

[49] Liu T., Dai A., Lu J., Yuan Y., Xiao Y., Yu L., Li M., Gim J., Ma L., Liu J. (2019). Correlation between
manganese dissolution and dynamic phase stability in spinel-based
lithium-ion battery. Nat. Commun..

[50] Pieczonka N. P., Liu Z., Lu P., Olson K. L., Moote J., Powell B. R., Kim J.-H. (2013). Understanding transition-metal
dissolution behavior
in LiNi0. 5Mn1. 5O4 high-voltage spinel for lithium ion batteries. J. Phys. Chem. C.

[51] Ma S., Noguchi H., Yoshio M. (2001). Cyclic voltammetric
study on stoichiometric
spinel LiMn2O4 electrode at elevated temperature. J. Power Sources.

[52] Hestenes J. C., Sadowski J. T., May R., Marbella L. E. (2023). Transition
metal
dissolution mechanisms and impacts on electronic conductivity in composite
LiNi0. 5Mn1. 5O4 cathode films. ACS Materials
Au.

[53] Okudur F. U., D’Haen J., Vranken T., De Sloovere D., Verheijen M., Karakulina O., Abakumov A., Hadermann J., Van Bael M. K., Hardy A. (2018). Ti surface doping of LiNi 0.5 Mn
1.5 O 4– δ positive electrodes for lithium ion batteries. RSC Adv..

[54] Çapraz Ö., Rajput S., White S., Sottos N. (2018). Strain evolution in
lithium manganese oxide electrodes. Experimental
Mechanics.

[55] Sun H., Hu A., Spence S., Kuai C., Hou D., Mu L., Liu J., Li L., Sun C., Sainio S. (2022). Tailoring
disordered/ordered
phases to revisit the degradation mechanism of high-voltage LiNi0.
5Mn1. 5O4 spinel cathode materials. Adv. Funct.
Mater..

[56] Jafta C. J., Mathe M. K., Manyala N., Roos W. D., Ozoemena K. I. (2013). Microwave-assisted
synthesis of high-voltage nanostructured LiMn1. 5Ni0. 5O4 spinel:
tuning the Mn3+ content and electrochemical performance. ACS Appl. Mater. Interfaces.

[57] Zeng C., Fan F., Zheng R., Wang X., Tian G., Liu S., Liu P., Wang C., Wang S., Shu C. (2024). Structure and charge
regulation strategy enabling superior cycling stability of Ni-rich
cathode materials. ACS Appl. Mater. Interfaces.

[58] Zeng C., Zheng R., Cao Y., Fan F., Huang Y., Zhang Y., Xiao H., Shu C., Zhang B. (2026). Gradient Micro-Structure
Design Enabling Mechanochemically Durable Single-Crystalline Ni-Rich
Layered Oxides for Advanced Lithium-Ion Batteries. Adv. Funct. Mater..

[59] Fan F., Zheng R., Zeng C., Xu H., Wang X., Tian G., Wang S., Wang C., Liu P., Shu C. (2025). Synergistically dissipating the local strain and restraining lattice
oxygen escape by fine-tuning of microstructure enabling Ni-rich cathodes
with superior cyclabilities. Journal of Energy
Chemistry.

[60] Zeng C., Zheng R., Fan F., Wang X., Tian G., Liu S., Liu P., Wang C., Wang S., Shu C. (2024). Phase compatible
surface engineering to boost the cycling stability of single-crystalline
Ni-rich cathode for high energy density lithium-ion batteries. Energy Storage Materials.

[61] Sharma V., Bhardwaj G., Mahendran N., Preetham K. B. A., Nukala P., Aetukuri N. P. B. (2025). Ti Doping Decreases
Mn and Ni Dissolution from High-Voltage
LiNi_0.5_Mn_1.5_O_4_ Cathodes. ACS Materials Au.

[62] Zhao J., Qian C., Yan G., Xu X., Lan C., Fu X. (2026). Enhancing Electrochemical Performance
of LiNi_0.5_Mn_1.5_O_4_ Cathode vis Synergistic
Ti/Zr Co-Doping. Journal of The electrochemical
Society.

