# Supplementary material for: Calcium Gradient-Doped LiNi0.5Mn1.5O4 Cathode for Long Cycle Life Lithium-Ion Batteries
Source: ACS Appl Energy Mater. 2026 Apr 23;9(9):5685–99. doi: 10.1021/acsaem.6c00247 (PMC13169349; doi:10.1021/acsaem.6c00247)
Supplement: Supplementary file 1 [file ae6c00247_si_001.pdf]

(Supporting information)

# Calcium Gradient-Doped $LiNi_{0.5}Mn_{1.5}O_4$ Cathode for Long Cycle Life Lithium-Ion Batteries

*Jie Xiong[a], Emmanuel Kornyo[a], Bingyao Zhou[a], Kevin Mathew[a], Guoxin Zhang[a], Wenquan Lu[b], Zhi Mei[c], Qingliu Wu[a,]**

a- Department of Chemical and Paper Engineering, Western Michigan University, 4601 Campus Drive, Kalamazoo, Michigan, 49008-5462, U. S. A.

b- Chemical Science and Engineering Division, Argonne National Laboratory, 9700 South Cass Ave., Lemont, Illinois, 60439-4837, U. S. A.

c- Lumigen Instrument Center, Wayne State University, 5101 Cass Ave, Detroit, Michigan, 48202, U. S. A.

* Correspondence should be addressed to:

Qingliu Wu, email: qingliu.wu@wmich.edu; Phone: 269-276-3998; Fax: 269-276-3501

**Figure S1**. Electron diffraction of Ca uniform 0.1 LNMO cathode.

**Figure S2**. The specific capacity of Ca gradient-doped LNMO cathodes as a function of current density. For comparison, the rate capability of undoped and Ca 0.05 uniform-doped LNMO cathodes was also shown here.

**Figure S3**. The voltage profiles of Ca gradient-doped LNMO electrodes at 2 C (a) and 10 C (b) during the discharge processes. For comparison, the voltage profiles of undoped and Ca 0.05 uniform-doped LNMO cathodes were also shown here.

**Figure S4**. Cycling performance of undoped and Ca gradient-doped LNMO at 55 ℃.

**Figure S5**. Discharge voltage profiles (a) and differential capacity (dQ/dV) profiles (b) of Ca 0.05 gradient-doped LNMO at selected cycles.

**Figure S6**. Coulombic efficiencies of undoped, Ca uniform 0.05, and Ca gr 0.05 LNMO during cycling.

**Table S1**. The peak intensity ratio I(400)/I(111) of undoped and Ca gradient-doped LNMO cathodes.

**Table S2**. Summary of formation data for LNMO cathode samples. Theoretical voltage plateau of 4.7 V is used to calculate polarization of $Ni^{2+}/Ni^{3+}$ plateau.

**Table S3**. Capacity contribution from the low-voltage plateau of LNMO cathodes during the third formation cycles.

**Table S4**. Summary of discharge capacities of LNMO cathodes at different C-rates.

**Table S5**. Summary of capacity retentions of LNMO cathodes at different C-rates.

**Table S6**. Summary of LNMO cell voltages at 50% SOC and ΔV at different C-rates.

**Table S7**. Anodic and cathodic peak separations (ΔV) for the $Ni^{2+}/Ni^{3+}$ and $Ni^{3+}/Ni^{4+}$ redox couples of LNMO cathodes.

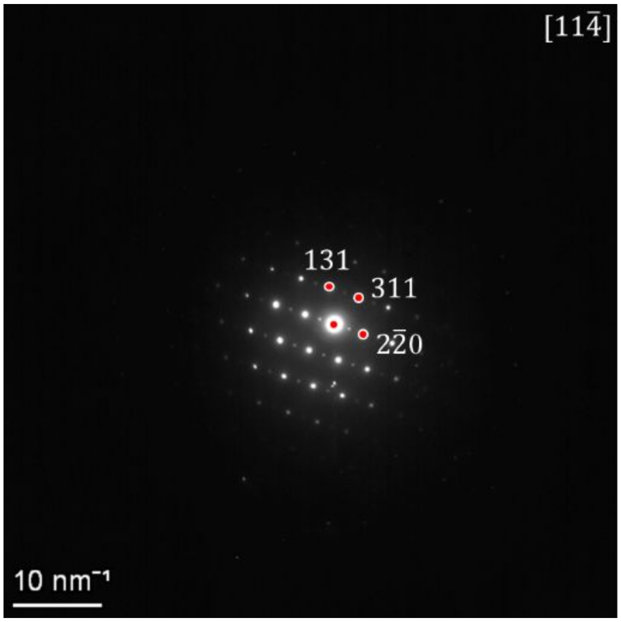


Figure S1. Electron diffraction of Ca uniform 0.1 LNMO cathode.

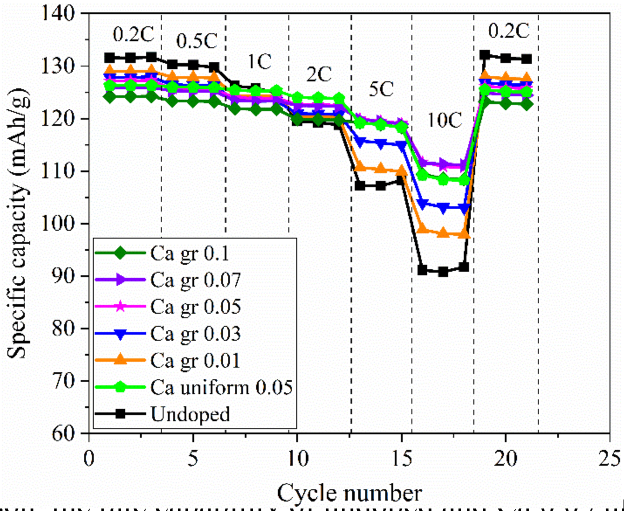


**Figure S2.** The specific [illegible] as a function of current density. For comparison, the rate capability of undoped and Ca 0.05 uniform-doped LNMO cathodes was also shown here.

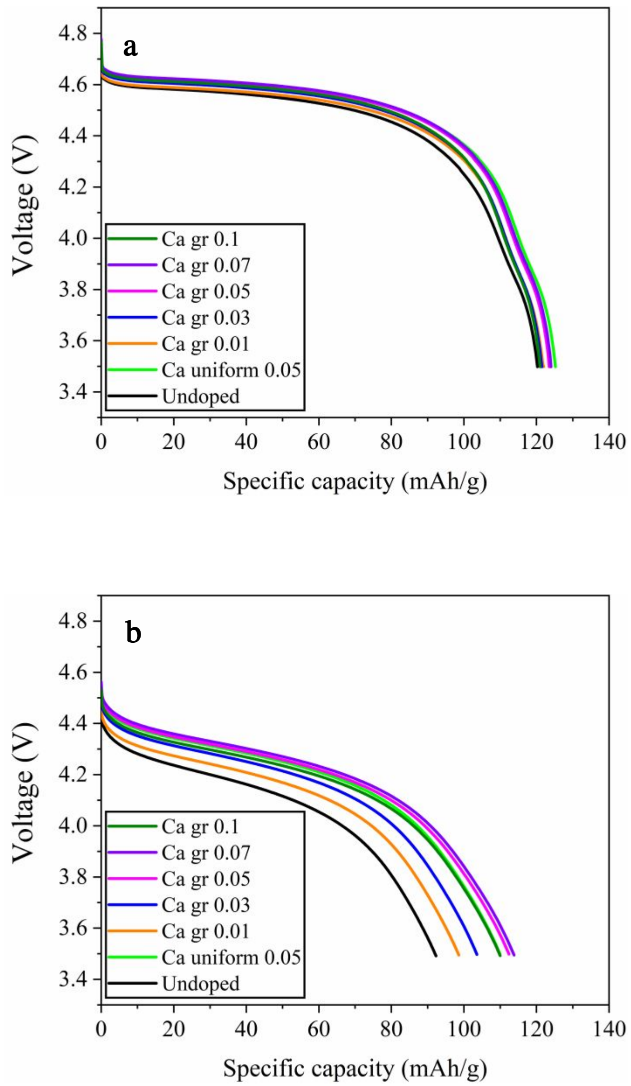


**Figure S3**. The voltage profiles of Ca gradient-doped LNMO electrodes at 2 C (a) and 10 C (b) during the discharge processes. For comparison, the voltage profiles of undoped and Ca 0.05 uniform-doped LNMO cathodes were also shown here.

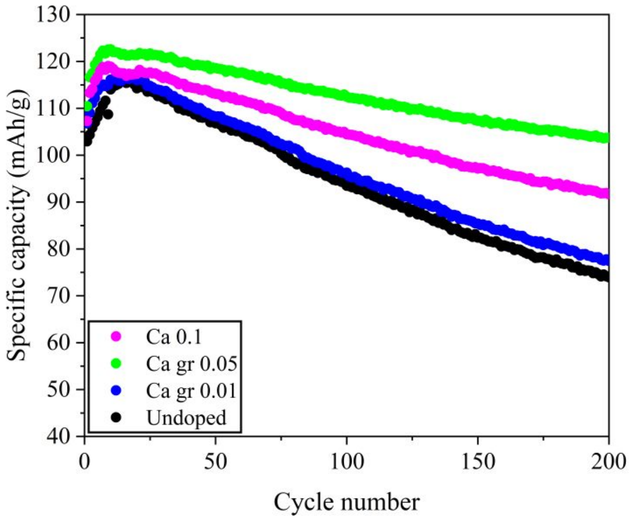


**Figure S4**. Cycling performance of undoped and Ca gradient-doped LNMO at 55 ℃.

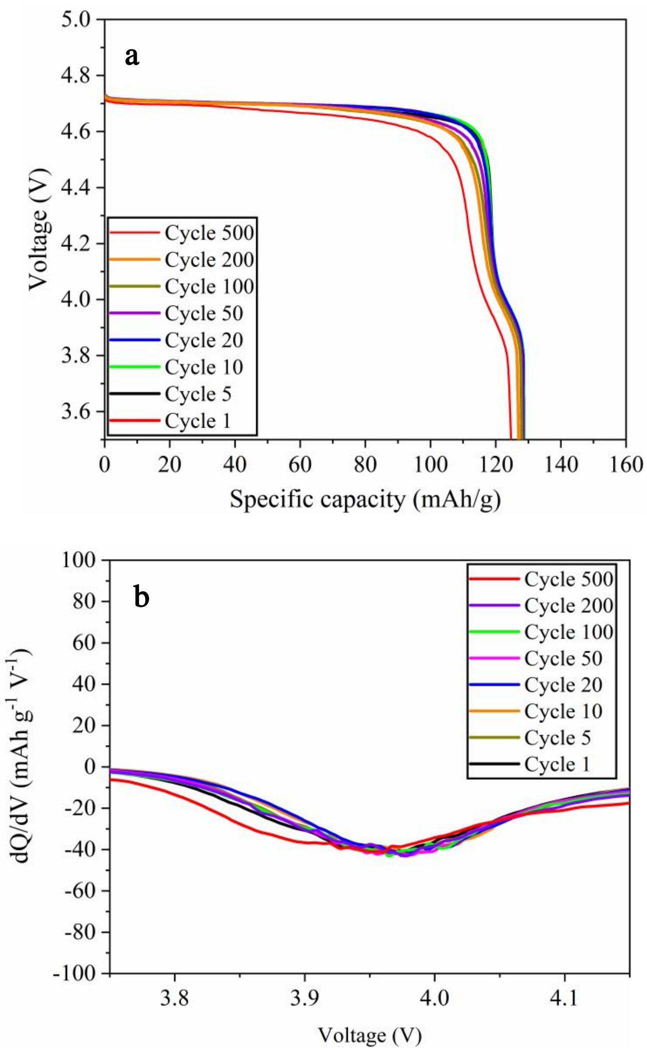


**Figure S5**. Discharge voltage profiles (a) and differential capacity (dQ/dV) profiles (b) of Ca 0.05 gradient-doped LNMO at selected cycles.

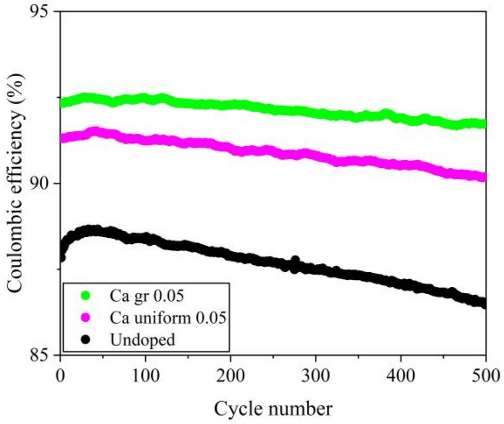


**Figure S6**. Coulombic efficiencies of undoped, Ca uniform 0.05, and Ca gr 0.05 LNMO during cycling.

**Table S1**. The peak intensity ratio I(400)/I(111) of undoped and Ca gradient-doped LNMO cathodes.

| LNMO Cathodes | I(400)/I(111) |
|---|---|
| Undoped | 0.54 |
| Ca uniform 0.05 | 0.54 |
| Ca gr 0.01 | 0.53 |
| Ca gr 0.03 | 0.49 |
| Ca gr 0.05 | 0.40 |
| Ca gr 0.07 | 0.38 |

**Table S2**. Summary of formation data for LNMO cathode samples. Theoretical voltage plateau of 4.7 V is used to calculate polarization of $Ni^{2+}/Ni^{3+}$ plateau.

| LNMO Cathodes | $3^{rd}$ Discharge capacity (mAh/g) | Coulombic efficiency (%) | $Ni^{2+}/Ni^{3+}$ Polarization $\Delta V$ (V) |
|---|---|---|---|
| Undoped | 131.4 | 87.9 | 0.0204 |
| Ca uniform 0.05 | 126.3 | 91.6 | 0.0105 |
| Ca gr 0.01 | 130.7 | 87.9 | 0.0149 |
| Ca gr 0.03 | 128.5 | 91.2 | 0.0107 |
| Ca gr 0.05 | 128.1 | 94.1 | 0.0104 |
| Ca gr 0.07 | 127.5 | 94.9 | 0.0102 |
| Ca gr 0.1 | 125.4 | 95.2 | 0.0104 |

**Table S3**. Capacity contribution from the low-voltage plateau of LNMO cathodes during the third formation cycles.

| Sample | $Mn^{3+}$ plateau (3.8-4.2V, mAh/g) | $Mn^{3+}$ plateau fraction (%) | Total discharge capacity (mAh/g) |
|---|---|---|---|
| Undoped | 12.1 | 9.2 | 131.4 |
| Ca uniform 0.05 | 7.7 | 6.1 | 126.3 |
| Ca gr 0.01 | 11.4 | 8.8 | 130.5 |
| Ca gr 0.03 | 10.4 | 8.1 | 128.4 |
| Ca gr 0.05 | 9.4 | 7.3 | 128.0 |
| Ca gr 0.07 | 8.4 | 6.6 | 127.5 |
| Ca gr 0.1 | 7.6 | 6.1 | 125.5 |

**Table S4**. Summary of discharge capacities of LNMO cathodes at different C-rates.

| LNMO Cathodes | 0.2 C | 2 C | 5 C | 10 C |
|---|---|---|---|---|
| Undoped | 131.6 | 119.5 | 107.2 | 91.1 |
| Ca uniform 0.05 | 126.3 | 124.0 | 119.2 | 109.2 |
| Ca gr 0.01 | 129.0 | 120.4 | 110.7 | 98.9 |
| Ca gr 0.03 | 127.8 | 120.9 | 115.7 | 103.9 |
| Ca gr 0.05 | 127.2 | 122.7 | 119.9 | 111.7 |
| Ca gr 0.07 | 125.9 | 122.5 | 119.8 | 111.5 |
| Ca gr 0.10 | 124.2 | 119.9 | 119.2 | 109.4 |

**Table S5**. Summary of capacity retentions of LNMO cathodes at different C-rates.

| LNMO Cathodes | 0.2C (%) | 2C (%) | 5C (%) | 10C (%) |
|---|---|---|---|---|
| Undoped | 100.0 | 90.8 | 81.5 | 69.3 |
| Ca uniform 0.05 | 100.0 | 98.2 | 94.4 | 86.5 |
| Ca gr 0.01 | 100.0 | 93.3 | 85.8 | 76.6 |
| Ca gr 0.03 | 100.0 | 94.6 | 90.5 | 81.3 |
| Ca gr 0.05 | 100.0 | 96.5 | 94.3 | 87.9 |
| Ca gr 0.07 | 100.0 | 97.3 | 95.2 | 88.6 |
| Ca gr 0.10 | 100.0 | 96.5 | 96.0 | 88.1 |

**Table S6**. Summary of LNMO cell voltages at 50% SOC and ΔV at different C-rates.

| LNMO Cathodes | V@50% (V, @0.2C) | V@50% (V, @2C) | V@50% (V, @10C) | ΔV (V, 0.2C→2C) | ΔV (V, 0.2C→10C) |
|---|---|---|---|---|---|
| Undoped | 4.701 | 4.527 | 4.135 | 0.174 | 0.566 |
| Ca uniform 0.05 | 4.702 | 4.564 | 4.232 | 0.138 | 0.470 |
| Ca gr 0.01 | 4.703 | 4.538 | 4.169 | 0.165 | 0.534 |
| Ca gr 0.03 | 4.703 | 4.554 | 4.206 | 0.149 | 0.497 |
| Ca gr 0.05 | 4.704 | 4.568 | 4.236 | 0.136 | 0.468 |
| Ca gr 0.07 | 4.705 | 4.572 | 4.247 | 0.133 | 0.458 |
| Ca gr 0.1 | 4.703 | 4.561 | 4.216 | 0.142 | 0.487 |

**Table S7**. Anodic and cathodic peak separations (ΔV) for the $Ni^{2+}/Ni^{3+}$ and $Ni^{3+}/Ni^{4+}$ redox couples of LNMO cathodes.

| LNMO Cathodes | ΔV (V, $Ni^{2+}/Ni^{3+}$ redox) | ΔV (V, $Ni^{3+}/Ni^{4+}$ redox) |
|---|---|---|
| Undoped | 0.272 | 0.238 |
| Ca uniform 0.05 | 0.254 | 0.230 |
| Ca gr 0.05 | 0.234 | 0.200 |
| Ca gr 0.1 | 0.260 | 0.242 |
